# Atlas of prostate cancer heritability in European and African-American men pinpoints tissue-specific regulation

**DOI:** 10.1038/ncomms10979

**Published:** 2016-04-07

**Authors:** Alexander Gusev, Huwenbo Shi, Gleb Kichaev, Mark Pomerantz, Fugen Li, Henry W. Long, Sue A. Ingles, Rick A. Kittles, Sara S. Strom, Benjamin A. Rybicki, Barbara Nemesure, William B. Isaacs, Wei Zheng, Curtis A. Pettaway, Edward D. Yeboah, Yao Tettey, Richard B. Biritwum, Andrew A. Adjei, Evelyn Tay, Ann Truelove, Shelley Niwa, Anand P. Chokkalingam, Esther M. John, Adam B. Murphy, Lisa B. Signorello, John Carpten, M. Cristina Leske, Suh-Yuh Wu, Anslem J. M. Hennis, Christine Neslund-Dudas, Ann W. Hsing, Lisa Chu, Phyllis J. Goodman, Eric A. Klein, John S. Witte, Graham Casey, Sam Kaggwa, Michael B. Cook, Daniel O. Stram, William J. Blot, Rosalind A. Eeles, Douglas Easton, ZSofia Kote-Jarai, Ali Amin Al Olama, Sara Benlloch, Kenneth Muir, Graham G. Giles, Melissa C. Southey, Liesel M. Fitzgerald, Henrik Gronberg, Fredrik Wiklund, Markus Aly, Brian E. Henderson, Johanna Schleutker, Tiina Wahlfors, Teuvo L. J. Tammela, Børge G. Nordestgaard, Tim J. Key, Ruth C. Travis, David E. Neal, Jenny L. Donovan, Freddie C. Hamdy, Paul Pharoah, Nora Pashayan, Kay-Tee Khaw, Janet L. Stanford, Stephen N. Thibodeau, Shannon K. McDonnell, Daniel J. Schaid, Christiane Maier, Walther Vogel, Manuel Luedeke, Kathleen Herkommer, Adam S. Kibel, Cezary Cybulski, Dominika Wokolorczyk, Wojciech Kluzniak, Lisa Cannon-Albright, Craig Teerlink, Hermann Brenner, Aida K. Dieffenbach, Volker Arndt, Jong Y. Park, Thomas A. Sellers, Hui-Yi Lin, Chavdar Slavov, Radka Kaneva, Vanio Mitev, Jyotsna Batra, Amanda Spurdle, Judith A. Clements, Manuel R. Teixeira, Hardev Pandha, Agnieszka Michael, Paula Paulo, Sofia Maia, Andrzej Kierzek, The PRACTICAL consortium, Margaret Cook, Michelle Guy, Koveela Govindasami, Daniel Leongamornlert, Emma J. Sawyer, Rosemary Wilkinson, Edward J. Saunders, Malgorzata Tymrakiewicz, Tokhir Dadaev, Angela Morgan, Cyril Fisher, Steve Hazel, Naomi Livni, Artitaya Lophatananon, John Pedersen, John L. Hopper, Jan Adolfson, Paer Stattin, Jan-Erik Johansson, Carin Cavalli-Bjoerkman, Ami Karlsson, Michael Broms, Anssi Auvinen, Paula Kujala, Liisa Maeaettaenen, Teemu Murtola, Kimmo Taari, Maren Weischer, Sune F. Nielsen, Peter Klarskov, Andreas Roder, Peter Iversen, Hans Wallinder, Sven Gustafsson, Angela Cox, Paul Brown, Anne George, Gemma Marsden, Athene Lane, Michael Davis, Wei Zheng, Lisa B. Signorello, William J. Blot, Lori Tillmans, Shaun Riska, Liang Wang, Antje Rinckleb, Jan Lubiski, Christa Stegmaier, Julio Pow-Sang, Hyun Park, Selina Radlein, Maria Rincon, James Haley, Babu Zachariah, Darina Kachakova, Elenko Popov, Atanaska Mitkova, Aleksandrina Vlahova, Tihomir Dikov, Svetlana Christova, Peter Heathcote, Glenn Wood, Greg Malone, Pamela Saunders, Allison Eckert, Trina Yeadon, Kris Kerr, Angus Collins, Megan Turner, Srilakshmi Srinivasan, Mary-Anne Kedda, Kimberly Alexander, Tracy Omara, Huihai Wu, Rui Henrique, Pedro Pinto, Joana Santos, Joao Barros-Silva, David V. Conti, Demetrius Albanes, Christine Berg, Sonja I. Berndt, Daniele Campa, E. David Crawford, W. Ryan Diver, Susan M. Gapstur, J. Michael Gaziano, Edward Giovannucci, Robert Hoover, David J. Hunter, Mattias Johansson, Peter Kraft, Loic Le Marchand, Sara Lindström, Carmen Navarro, Kim Overvad, Elio Riboli, Afshan Siddiq, Victoria L. Stevens, Dimitrios Trichopoulos, Paolo Vineis, Meredith Yeager, Gosia Trynka, Soumya Raychaudhuri, Frederick R. Schumacher, Alkes L. Price, Matthew L. Freedman, Christopher A. Haiman, Bogdan Pasaniuc

**Affiliations:** 1 Program in Genetic Epidemiology and Statistical Genetics, Harvard T.H. Chan School of Public Health, Boston, Massachusetts 02115, USA; 2 Broad Institute of Harvard and MIT, Cambridge, Massachusetts 02142, USA; 3 Bioinformatics Interdepartmental Program, University of California Los Angeles, Los Angeles, California 90095, USA; 4 Department of Medical Oncology, Dana-Farber Cancer Institute and Harvard Medical School, Boston, Massachusetts 02115, USA; 5 Center for Functional Cancer Epigenetics, Dana-Farber Cancer Institute, Boston, Massachusetts 02115, USA; 6 Department of Biostatistics and Computational Biology, Dana-Farber Cancer Institute, Boston, Massachusetts 02115, USA; 7 Department of Preventative Medicine, Keck School of Medicine, University of Southern California/Norris Comprehensive Cancer Center, Los Angeles, California 90033, USA; 8 University of Arizona College of Medicine and University of Arizona Cancer Center, Tucson, Arizona 85721, USA; 9 Department of Epidemiology, University of Texas M.D. Anderson Cancer Center, Houston, Texas 77030, USA; 10 Department of Public Health Sciences, Henry Ford Hospital, Detroit, Michigan 48202, USA; 11 Department of Preventive Medicine, Stony Brook University, Stony Brook, New York 11794, USA; 12 James Buchanan Brady Urological Institute, Johns Hopkins Hospital and Medical Institution, Baltimore, Maryland 21205, USA; 13 Division of Epidemiology, Department of Medicine, Vanderbilt Epidemiology Center, Vanderbilt University School of Medicine, Nashville, Tennessee 37232, USA; 14 Department of Urology, University of Texas M.D. Anderson Cancer Center, Houston, Texas 77030, USA; 15 Korle Bu Teaching Hospital, Accra, Ghana; 16 University of Ghana Medical School, Accra, Ghana; 17 Westat, Rockville, Maryland 20850, USA; 18 School of Public Health, University of California, Berkeley, California 94720, USA; 19 Cancer Prevention Institute of California, Fremont, California 94538, USA; 20 Stanford University School of Medicine and Stanford Cancer Institute, Palo Alto, California 94305, USA; 21 Department of Urology, Northwestern University, Chicago, Illinois 60611, USA; 22 International Epidemiology Institute, Rockville, Maryland 20850, USA; 23 The Translational Genomics Research Institute, Phoenix, Arizona 85004, USA; 24 Chronic Disease Research Centre and Faculty of Medical Sciences, University of the West Indies, Bridgetown, Barbados; 25 SWOG Statistical Center, Fred Hutchinson Cancer Research Center, Seattle, Washington 98109, USA; 26 Glickman Urological & Kidney Institute, Cleveland Clinic, Cleveland, Ohio 44195, USA; 27 Department of Epidemiology and Biostatistics, University of California, San Francisco, San Francisco, California 94118, USA; 28 Institute for Human Genetics, University of California, San Francisco, San Francisco, California 94118, USA; 29 Department of Surgery, Makerere University College of Health Sciences, Kampala 94118, Uganda; 30 Division of Cancer Epidemiology and Genetics, National Cancer Institute, Bethesda, Maryland 20892, USA; 31 The Institute of Cancer Research, Sutton SM2 5NG, UK; 32 Royal Marsden National Health Service (NHS) Foundation Trust, London and Sutton, UK; 33 Centre for Cancer Genetic Epidemiology, Department of Public Health and Primary Care, University of Cambridge, Strangeways Laboratory, Worts Causeway, Cambridge CB1 8RN, UK; 34 Institute of Population Health, University of Manchester, Manchester M13 9PL, UK; 35 Warwick Medical School, University of Warwick, Coventry CV4 7AL, UK; 36 Cancer Epidemiology Centre, The Cancer Council Victoria, 615 St Kilda Road, Melbourne, Victoria 3004, Australia; 37 Centre for Epidemiology and Biostatistics, Melbourne School of Population and Global Health, The University of Melbourne, Victoria 3004, Australia; 38 Genetic Epidemiology Laboratory, Department of Pathology, The University of Melbourne, Grattan Street, Parkville, Victoria 3010, Australia; 39 Department of Medical Epidemiology and Biostatistics, Karolinska Institute, Stockholm 171 77, Sweden; 40 Department of Clinical Sciences at Danderyds Hospital, Stockholm 171 77, Sweden; 41 Department of Preventive Medicine, Keck School of Medicine, University of Southern California/Norris Comprehensive Cancer Center, Los Angeles, California 90007, USA; 42 Department of Medical Biochemistry and Genetics Institute of Biomedicine Kiinamyllynkatu 10, University of Turku, Turku FI-20014, Finland; 43 BioMediTech, University of Tampere and FimLab Laboratories, Tampere 33200, Finland; 44 Department of Urology, Tampere University Hospital and Medical School, University of Tampere, Tampere 33200, Finland; 45 Department of Clinical Biochemistry, Herlev Hospital, Copenhagen University Hospital, Herlev Ringvej 75, Herlev DK-2730, Denmark; 46 Faculty of Health and Medical Sciences, University of Copenhagen, Copenhagen 1165, Densmark; 47 Cancer Epidemiology, Nuffield Department of Population Health; University of Oxford, Oxford OX3 7LF, UK; 48 University of Cambridge, Department of Oncology, Addenbrooke's Hospital, Box 279, Hills Road, Cambridge CB2 0QQ; 49 Cancer Research UK Cambridge Research Institute, Li Ka Shing Centre, Cambridge, UK; 50 School of Social and Community Medicine, University of Bristol, Canynge Hall, 39 Whatley Road, Bristol BS8 2PS, UK; 51 Department of Public Health, Section for Epidemiology, Aarhus University, Aarhus OX1 3PN, Denmark; 52 Faculty of Medical Science, University of Oxford, John Radcliffe Hospital, Oxford OX1 3PN, UK; 53 Centre for Cancer Genetic Epidemiology, Department of Oncology, University of Cambridge, Strangeways Laboratory, Worts Causeway, Cambridge CB1 8RN, UK; 54 University College London, Department of Applied Health Research, 1-19 Torrington Place, London WC1E 7HB, UK; 55 Clinical Gerontology Unit, University of Cambridge, Cambridge CB1 8RN, UK; 56 Division of Public Health Sciences, Fred Hutchinson Cancer Research Center, Seattle, Washington 98109-1024, USA; 57 Department of Epidemiology, School of Public Health, University of Washington, Seattle, Washington 98109, USA; 58 Mayo Clinic, Rochester, Minnesota 55905, USA; 59 Institute of Human Genetics, University Hospital Ulm, 89081 Ulm, Germany; 60 Department of Urology, University Hospital Ulm, 89081 Ulm, Germany; 61 Department of Urology, Klinikum rechts der Isar der Technischen Universitaet Muenchen, 81675 Munich, Germany; 62 Division of Urologic Surgery, Brigham and Womens Hospital, Dana-Farber Cancer Institute, 75 Francis Street, Boston, Massachusetts 02115, USA; 63 International Hereditary Cancer Center, Department of Genetics and Pathology, Pomeranian Medical University, Szczecin, Poland; 64 Division of Genetic Epidemiology, Department of Medicine, University of Utah School of Medicine, Salt Lake City, Utah 84132, USA; 65 George E. Wahlen Department of Veterans Affairs Medical Center, Salt Lake City, Utah 84132, USA; 66 Division of Clinical Epidemiology and Aging Research, German Cancer Research Center (DKFZ), Heidelberg 69120, Germany; 67 German Cancer Consortium (DKTK), Heidelberg 69120, Germany; 68 Department of Cancer Epidemiology, Moffitt Cancer Center, 12902 Magnolia Drive, Tampa, Florida 33612, USA; 69 Biostatistics Program, Moffitt Cancer Center, 12902 Magnolia Drive, Tampa, Florida 33612, USA; 70 Department of Urology and Alexandrovska University Hospital, Medical University, Sofia 1431, Bulgaria; 71 Department of Medical Chemistry and Biochemistry, Molecular Medicine Center, Medical University, Sofia, 2 Zdrave Str., Sofia 1431, Bulgaria; 72 Australian Prostate Cancer Research Centre-Qld, Institute of Health and Biomedical Innovation and School of Biomedical Science, Queensland University of Technology, Brisbane, Queensland 4000, Australia; 73 Molecular Cancer Epidemiology Laboratory, Queensland Institute of Medical Research, Brisbane, Queensland 4000, Australia; 74 Department of Genetics, Portuguese Oncology Institute, Porto 4200, Portugal; 75 Biomedical Sciences Institute (ICBAS), University of Porto, Porto 4200, Portugal; 76 The University of Surrey, Guildford, Surrey GU2 7XH, UK; 77 Department of Preventive Medicine, Norris Cancer Center, University of Southern California Keck School of Medicine, Los Angeles, California 90033, USA; 78 Nutritional Epidemiology Branch, Division of Cancer Epidemiology and Genetics, National Cancer Institute, US National Institute of Health, Bethesda, Maryland 20892, USA; 79 Department of Radiation Oncology and Molecular Radiation Sciences, Johns Hopkins Medicine, Baltimore, Maryland 21287, USA; 80 Genomic Epidemiology Group, German Cancer Research Center (DKFZ), 69121 Heidelberg, Germany; 81 Urologic Oncology, University of Colorado at Denver Health Sciences Center, Denver, Colorado 80230, USA; 82 Epidemiology Research Program, American Cancer Society, Atlanta, Georgia 30303, USA; 83 Department of Medicine, Harvard Medical School, Boston, Massachusetts 02115, USA; 84 Division of Aging, Brigham and Women's Hospital, Boston, Massachusetts 02115, USA; 85 Department of Nutrition, Harvard School of Public Health, Boston, Massachusetts 02115, USA; 86 International Agency for Research on Cancer, Lyon 69008, France; 87 Department of Surgical and Perioperative Sciences, Urology and Andrology, Umeå University, Umeå 907 36, Sweden; 88 Department of Biostatistics, Harvard School of Public Health, Boston, Massachusetts 02115, USA; 89 Epidemiology Program, University of Hawaii Cancer Center, Honolulu, Hawaii 96813, USA; 90 Department of Epidemiology, Regional Health Authority, Murcia 30009, Spain; 91 CIBER Epidemiología y Salud Pública (CIBERESP), Barcelona 28029, Spain; 92 Department of Epidemiology and Biostatistics, School of Public Health, Imperial College London, London SW7 2AZ, UK; 93 Department of Genomics of Common Disease, School of Public Health, Imperial College London, London SW7 2AZ, UK; 94 Bureau of Epidemiologic Research, Academy of Athens, Athens 106 79, Greece; 95 Hellenic Health Foundation, Athens 106 79, Greece; 96 HuGeF Foundation, Torino 10126, Italy; 97 School of Public Health, Imperial College London, London SW7 2AZ, UK; 98 Divisions of Genetics and Rheumatology, Department of Medicine, Brigham and Women's Hospital and Harvard Medical School, Boston, Massachusetts, USA; 99 Wellcome Trust Sanger Institute, Wellcome Trust Genome Campus, Cambridge CB10 1SA, UK; 100 Institute of Inflammation and Repair, University of Manchester, Manchester M13 9PT, UK; 101 Departments of Pathology and Laboratory Medicine, University of California Los Angeles, Los Angeles, California, USA; 102 Department of Human Genetics, University of California Los Angeles, Los Angeles, California 90095, USA; 103 Tissupath Pty Ltd., Melbourne,Victoria 3122, Australia; 104 Department of Epidemiology, School of Health Sciences, University of Tampere, Tampere 33014, Finland; 105 Fimlab Laboratories, Tampere University Hospital, Tampere, Finland; 106 Finnish Cancer Registry, Helsinki, Finland; 107 School of Medicine, University of Tampere, Tampere, Finland; 108 Department of Urology, Tampere University Hospital, Tampere, Finland; 109 Department of Urology, Helsinki University Central Hospital and University of Helsinki, Helsinki 00100, Finland; 110 Department of Urology, Herlev Hospital, Copenhagen University Hospital, Herlev Ringvej 75, Herlev DK-230, Denmark; 111 Copenhagen Prostate Cancer Center, Department of Urology, Rigshospitalet, Copenhagen University Hospital, Tagensvej 20, 7521, Copenhagen DK-2200, Denmark; 112 Department of Epidemiology and Biostatistics, School of Public Health, Imperial College, London SW7 2AZ, UK; 113 CR-UK/YCR Sheffield Cancer Research Centre, University of Sheffield, Sheffield S10 2TN, UK; 114 Division of Epidemiology, Department of Medicine, Vanderbilt University Medical Center, 2525 West End Avenue, Suite 800, Nashville, Tennessee 37232, USA; 115 National Cancer Institute, NIH, 9609 Medical Center Drive, Suite 2W-172, MSC 9712, Bethesda, MD 20892-9712 (mail), Rockville, Maryland 20850 (FedEx/Courier), USA; 116 International Epidemiology Institute, 1555 Research Blvd., Suite 550, Rockville, Maryland 20850, USA; 117 Division of Epidemiology, Department of Medicine, Vanderbilt Epidemiology Center, Vanderbilt-Ingram Cancer Center, Vanderbilt University School of Medicine, Nashville, Tennessee 37232, USA; 118 Saarland Cancer Registry, Saarbrücken 66119, Germany; 119 The University of Surrey, Guildford, Surrey GU2 7XH

## Abstract

Although genome-wide association studies have identified over 100 risk loci that explain ∼33% of familial risk for prostate cancer (PrCa), their functional effects on risk remain largely unknown. Here we use genotype data from 59,089 men of European and African American ancestries combined with cell-type-specific epigenetic data to build a genomic atlas of single-nucleotide polymorphism (SNP) heritability in PrCa. We find significant differences in heritability between variants in prostate-relevant epigenetic marks defined in normal versus tumour tissue as well as between tissue and cell lines. The majority of SNP heritability lies in regions marked by H3k27 acetylation in prostate adenoc7arcinoma cell line (LNCaP) or by DNaseI hypersensitive sites in cancer cell lines. We find a high degree of similarity between European and African American ancestries suggesting a similar genetic architecture from common variation underlying PrCa risk. Our findings showcase the power of integrating functional annotation with genetic data to understand the genetic basis of PrCa.

## Abstract (web summary)

Over one hundred loci have been identified to be associated with the familial risk of prostate cancer but the functional effects are poorly understood. Here the authors use single-nucleotide variant and epigentic data to show an underlying genetic architecture marked by histone modification.

## Graphical abstract

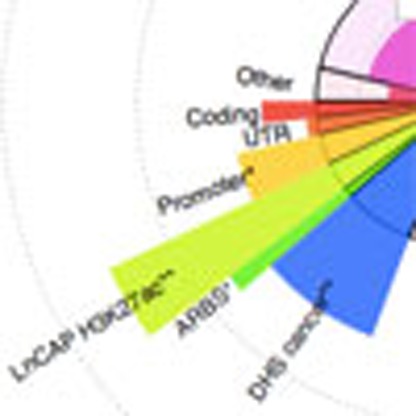

Family history is a well-established risk factor for prostate cancer (PrCa), which has an estimated heritability of 58%—one of the highest across common cancers^1^. Genome-wide association studies (GWAS) have been particularly successful in identifying over 100 risk loci that capture ∼33% of the estimated familial risk^2^. Although most of the GWAS PrCa variants overlap prostate-specific regulatory elements (for example, androgen receptor-binding sites (ARBS))^2^,^3^,^4^,^5^,^6^,^7^,^8^, a quantification of the contribution of genetic variation from various chromatin marks to PrCa risk is currently lacking.

Recent work form the ENCODE/ROADMAP consortia^9^ has shown that a large fraction of the genome plays a role in at least one biochemical event, in at least one tissue. Although this functional atlas of the human genome has greatly enhanced our understanding of regulatory elements, such functional elements are often tissue specific^10^,^11^ making their interpretability in the context of PrCa risk challenging. Existing studies that have integrated PrCa GWAS findings with tissue-specific functional annotations have relied only on the GWAS significant variants (∼100 in the most recent study) or single-nucleotide polymorphisms (SNPs) tagging them^2^,^7^, thus ignoring loci that do not reach genome-wide significance. Recent methodological advances have shown that the entire polygenic architecture of common traits can be interrogated using variance components across all assayed SNPs (typed and/or imputed) to increase power for detecting trait-specific functional annotations^12^. In addition to offering superior performance relative to methods that evaluate only GWAS SNPs, the variance components methods also allow for comparison of estimates across different studies and sample sizes. This is because variance components yield an unbiased estimate (under standard assumptions) of SNP heritability 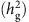
—the variance in trait explained by SNPs that reside within elements of a given functional category^12^,^13^,^14^,^15^.

Here, we use targeted and genome-wide SNP array data from 59,089 male PrCa cases and controls of European (BPC3 (ref. 16) and iCOGS (ref. 4), respectively, see Methods) and African American (AAPC (ref. 17), see Methods) ancestry to dissect the genetic risk of PrCa. We estimate the SNP heritability of previously implicated regulatory annotations^7^,^18^ and perform a broad analysis of 544 epigenetic marks from ENCODE/ROADMAP (ref. 9). Our approach interrogates the entire common polygenic architecture of PrCa while accounting for potential correlations between related functional categories. First, we find that SNPs near ARBS assayed in prostate tumour explain significantly more of the heritability of PrCa than ARBS SNPs assayed in prostate normal tissue. Second, we localize most of the heritability of PrCa to regions in the genome marked by three functional categories: (i) H3K27ac histone modifications in prostate adenocarcinoma cell lines (LNCaP; typically marking active enhancers^19^); (ii) androgen receptors in prostate tissue^18^; and (iii) DNase I hypersensitivity sites (DHS) in cancer cell lines. We replicate the LNCaP H3K27ac and DHS results across different ancestries and show that risk prediction from genome-wide SNP data is significantly improved with a predictor that incorporates the functional atlas as prior. Overall, our results suggest a similar genetic architecture from common variation of PrCa risk across men of European and African ancestry and highlight H3k27ac histone mark in LNCaP and ARBS in prostate tissue for follow-up studies of PrCa risk.

## Results

### Partitioning the genetic risk for prostate cancer

We analysed multiple functional annotations and quantified the fraction of variance in trait explained by SNPs that are localized within each functional class. Our approach models the phenotype (PrCa) of a set of individuals as being drawn from a multivariate normal distribution with variance components estimated based on genetic data (that is, SNPs) plus an environmental term (see Methods)^13^,^14^. For each functional category *i*, a genetic relationship matrix across all individuals is computed from all the SNPs residing in the given functional category to serve as a variance component. Multiple components are then jointly fitted using the restricted maximum likelihood (REML) as implemented in the GCTA software^14^ to estimate variance parameters 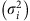 for each component. The SNP heritability for component *i* is then estimated as 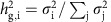, where the sum in the denominator is across all fitted components including the environmental term. Therefore, we view 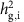
as an estimate of the variance in trait that can be explained by all the SNPs in the corresponding functional category with a linear model of the trait (that is, SNP heritability)^12^. We expect functional categories that are enriched with casual variants for PrCa to attain a higher estimated SNP heritability as compared with functional categories depleted of causal variants for PrCa. To focus our results on noncoding variation and account for potential confounders because of linkage disequilibrium (LD), we explicitly included coding and coding-proximal regulatory variation as ‘background' components whenever we quantified the effect of each functional annotation tested (see Methods).

The variance component model has previously been shown to yield robust estimates under the assumption that causal variants are typed and uniformly sampled from a given component^13^,^20^,^21^. Here, we perform additional simulations using the UK10K whole-genome sequence data to confirm the validity of this model for our data, and to assess how representative SNP estimates are of true underlying biology at common sequenced variants. The simulation framework uses real genotype data from the UK10K consortium to generate additive, polygenic phenotypes with a given heritability and then performs heritability estimation with the variance component model (see Methods). Although the UK10K data contains a much smaller set of individuals as the iCOGS data (3,047 versus 42,613 individuals, see Methods), it contains variation from whole-genome sequencing; this allows us to evaluate model performance by simulation when restricting to SNPs genotyped on the iCOGS platform. We focused on the LNCaP: H3k27ac annotation (which was most significant in our data, see below) to evaluate the multiple component models. Over thousands of simulations, we confirmed that the variance components approach correctly recovered the causal contribution to trait from a given functional category when causal variants were typed (Supplementary Table 1, see Methods). Under both null and enriched scenarios the estimates were unbiased and standard errors properly calibrated (Supplementary Table 1). For common sequenced variants not present on the iCOGS platform, relative estimates of noncoding enrichment/depletion were conservative, with the tagged effects distributed across the typed components (Supplementary Table 2). Deviations from the standard variance components model assumptions on the distribution of effect-sizes and ancestry-specific effects in African Americans yielded either well calibrated or conservative estimates of SNP heritability in the focal LNCaP: H3k27ac category (see Methods, Supplementary Tables 1–3).

Our primary functional analyses focus on the densely genotyped iCOGS sample (21,678 cases and 20,935 controls), whose large sample size allowed for highly accurate estimates of component-specific 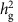. Although the iCOGS chip is custom built to oversample risk loci, it provides a broad coverage of the common variation genome wide^4^. To showcase the power of the variance components approach, we estimated the total SNP heritability of PrCa at 0.28 (s.e. 0.01) in the iCOGS data (not significantly different from the total SNP heritability estimate of 0.26 (s.e. 0.05) in the BPC3 data), a significant increase from the variance explained only by the known GWAS variants 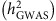 of 0.06 (s.e.m. 0.001) (see Methods; Supplementary Table 4). Interestingly, the total SNP heritability in the African American sample, which was genotyped on a different platform than iCOGS (see Methods), was estimated at 0.32 (s.e. 0.06) indicating a similar aggregate contribution of common variation to PrCa risk across the two ethnicities despite higher overall risk in African Americans^22^ (Supplementary Table 4).

### Enrichment at androgen receptor-binding sites in tumours

**Figure 1:**
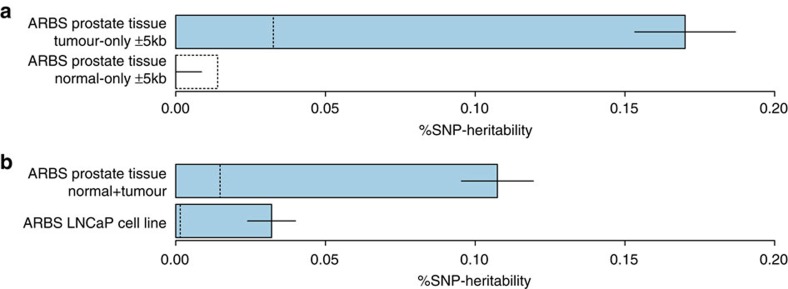
Functional partitioning for variants within ARBS for PrCa. Bars graphs detailing %SNP heritability estimates from two models of PrCa relevant functional annotations. (**a**) Joint comparison of variants within 5 kb of tumour-only and normal-only regions in the ARBS in prostate tissue (*P*=2.1 × 10^−19^ for difference by *Z*-test). (**b**) Estimates from ARBS in prostate tissue (no longer using a 5 kb flank) and ARBS in LNCaP cell lines^7^ (*P*=4.4 × 10^−7^ for difference). The null 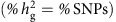 is labelled by the dashed lines. Error bars show analytical standard error of estimate.

We first focused on SNPs localized in the ARBS: an epigenetic profile causally implicated in prostate tumorigensis. In contrast to typical assays that focus on cell lines, the ARBS were defined by chromatin immunoprecipitation and high-throughput sequencing (ChIP-seq) directly in primary human tissue (seven normal and 13 tumour specimens)^18^. We observed that variants within 5 kb of tumour-specific ARBS explained 17.0% of the genome-wide 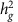 (s.e. 1.7%; *P*=2.6 × 10^−16^ by *Z*-test), whereas the variants near normal-specific ARBS explained 0.0% of the 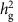 (s.e. 0.9%; *P*=0.11 by *Z*-test) (Fig. 1). The difference between these two groups was highly significant and demonstrates the importance of assaying functional marks in both normal and tumour tissues. We note that the 5 kb extension may also include other regulatory variants near the tumour/normal-specific ARBS (but not heritability from coding/untranslated region (UTR)/promoter variants, which were explicitly modelled, see Methods). Smaller flanking regions were also investigated but did not include enough markers for the variance components model to converge. We also quantified the proportion of SNP heritability explained directly by all ARBS variants (both normal and tumour without 5 kb flanks) at 10.7% of 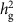; significantly different from the SNP heritability of ARBS variants assayed in prostate adenocarcinoma cancer cell line (LNCaP; 3.2% of 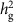) (*P*=4.4 × 10^−7^ for difference by *Z*-test) (Fig. 1). This difference is partially explained by the very low number of SNPs within cell line ARBS making their aggregate contribution small but not empowering us to place a strong bound on the enrichment. Overall, these findings highlight the increased complexity of ARBS in a sample of tissues as compared with the single LNCaP cell line.

### Identification of functional marks relevant to PrCa risk

**Figure 2:**
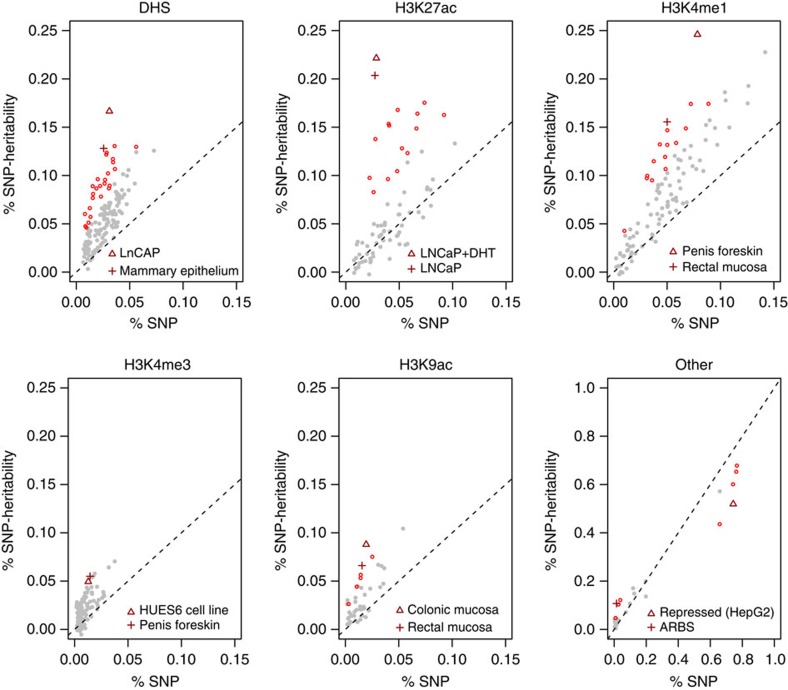
Functional partitioning of heritability across six main epigenetic classes. Each point corresponds to an estimate of % SNP heritability (*y* axis) from SNPs within a cell-type-specific functional annotation versus annotation size (%SNPs, *x* axis). Overall, 544 annotations were tested, and red points indicate significant deviations from the null of 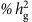 equal to %SNPs after accounting for all tests. The two most significant annotations in each class are shown with triangle/cross, respectively, and labelled in bottom right (see Supplementary Data for all annotations).

Next, we looked for marks that contribute to the heritability of PrCa across a broad spectrum of functional annotations without prior assumptions on relevance to disease. We investigated 544 epigenetic annotations spanning six major classes (DHS; H3k4me1; H3k4me3; H3k9ac; H3k27ac; and computationally predicted functional classes or ‘segmentations'^23^,^24^) averaging 101 cell types per class (see Methods). After accounting for multiple testing, we identified 82 annotations that exhibited statistically significant deviations in SNP heritability from what was expected based on the proportion of the genome covered by that particular annotation (see Fig. 2 and Supplementary Data).

**Figure 3:**
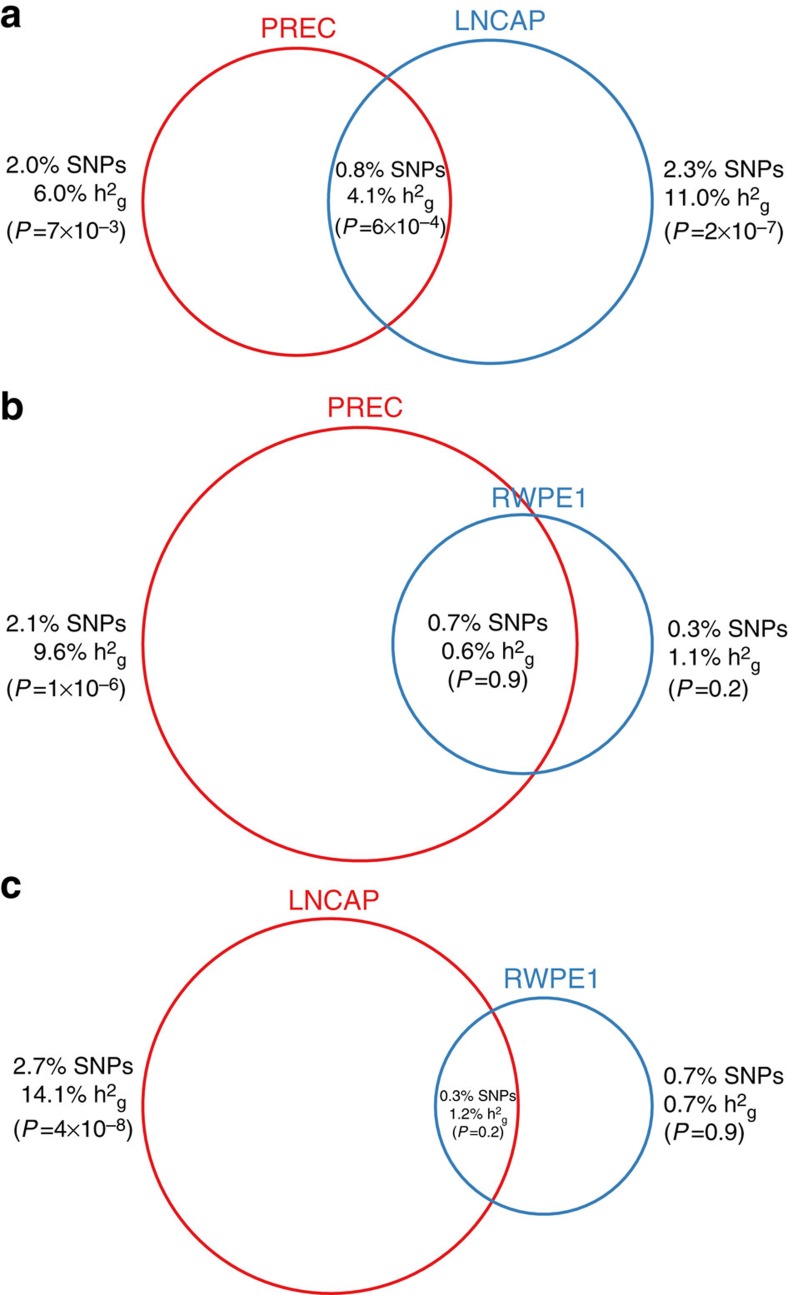
Pairwise analysis of DHS marks in three prostate cell types. Joint model from all pairs of DHS marks shown for: cancer cell line (LNCAP); normal prostate epithelial (PREC); and immortalized prostate epithelial (RWPE1). Circle size corresponds to % SNPs, with % SNP heritability and significance labelled. *P* value was computed for difference between 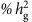 and %SNP, with bold representing significance after correcting for nine tests. The observed trend is LNCAP>PREC>RWPE1: (**a**)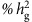 in LNCaP DHS was nominally significantly higher than PrEC (*P*=0.01); and 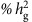 in LNCaP and PrEC was significantly higher than RWPE1 (**b**,**c**; *P*=1.5 × 10^−9^, *P*=1.2 × 10^−5^, respectively). All *P* values computed by *Z*-test using 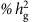 estimate and analytical standard error.

We first focused on 17 functional marks measured in the prostate, of which 14 were statistically significant (Supplementary Table 5). The single most significant enrichment was observed for H3k27ac marks in LNCaP (*P*=1 × 10^−32^ by *Z*-test), which localized 22% of the total 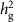 to the 2.9% of genotyped SNPs within the annotation. This was followed by variants in DHS marks in LNCaP (*P*=2 × 10^−18^ by *Z*-test; 16.7% of 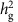 localized in 3.1% of genome). The DHS annotations allowed us to compare estimates across three major prostate cell lines: LNCaP; normal prostate epithelial (PrEC); and immortalized prostate epithelial (RWPE1) (overlapping by 25–50% with ARBS, Supplementary Fig. 1). We observed heritability explained by LNCaP DHS to be nominally significantly higher than PrEC (*P*=0.01 by *Z*-test); and both LNCaP and PrEC to be significantly higher than RWPE1 (*P*=1.5 × 10^−9^, *P*=1.2 × 10^−5^, respectively, by *Z*-test) (Fig. 3). More broadly, 10 out of 16 DHS marks measured in cancer cell lines were observed as significant, with colorectal cancer as the next most significant cancer (*P*=6.0 × 10^−10^ by *Z*-test; 9.4% of heritability localized in 2.0% of genome; Supplementary Data). H3k27ac in LNCaP remained the most significantly enriched mark across all 544 annotations (presented in detail in the Supplementary Data). The most depleted categories were repressed regions computationally predicted by Segway-chromHMM in HepG2 cells (*P*=1.3 × 10^−19^ by *Z*-test; 51.9% of 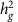 from 74.3% of SNPs; Supplementary Data), with similar levels of depletion in repressed regions from other cell types. These regions are typically associated with decreased gene expression and repressive histone marks^23^,^24^,^25^, further emphasizing the importance of active regulation.

**Figure 4:**
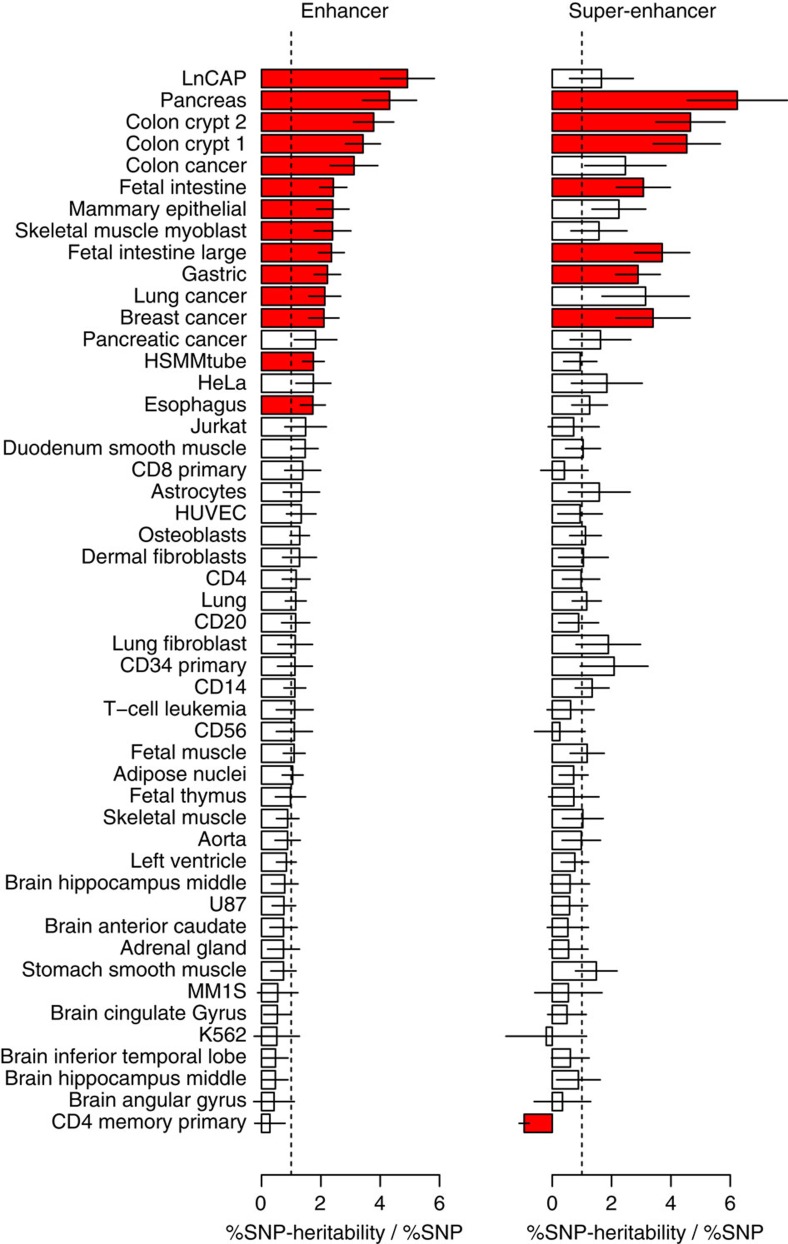
Comparison of enhancers and super enhancers across 49 cell types. Each bar represents the %SNP heritability 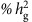/ %SNP for enhancers (left) and super enhancers (right) from a given cell type tested marginally. Red indicates significant difference from 1.0 (no enrichment) after accounting for 49 tests. Enhancer LNCAP is most significant, with other cancers also appearing significant and non-cancer tissues least significant. Error bars show analytical s.e. of estimate.

As H3k27ac typically marks active enhancers, we further evaluated variants with respect to their enhancer or ‘super'-enhancer status (large clusters of enhancers that are enriched for genes involved in cell identity^26^) (see Methods). We did not observe differences in average heritability explained by SNPs within the two marks across 49 cell lines (see Methods), with an average of 1.51 (1.47)-fold increase over random SNPs for enhancers (super enhancers) (Fig. 4). Surprisingly, we observed an individually significant difference only in LNCaP, with 4.9 (1.7)-fold enrichment at enhancers (super enhancers), in contrast to previous hypotheses^26^ (Fig. 4).

### Genomic functional atlas of prostate cancer SNP heritability

**Figure 5:**
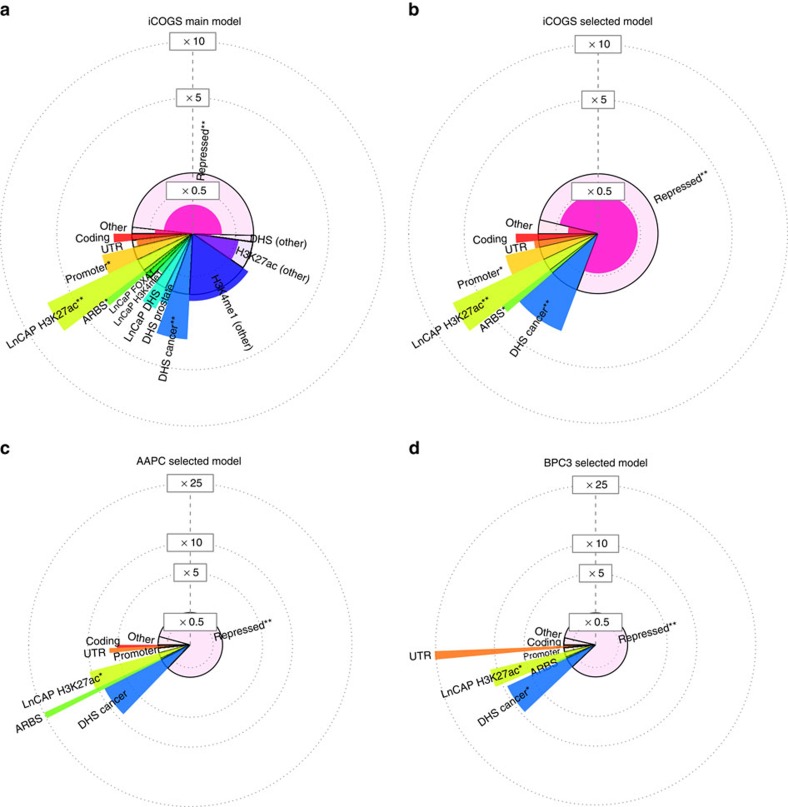
Partitioning of heritability across functional classes in prostate cancer. Visual representation of heritability enrichment in three studies **a**,**b**: iCOGS; **c**: AAPC; **d**: BPC3 (shown numerically in Table 1). Each subplot corresponds to an analysis of the listed joint model, with coloured slices representing the functional annotations evaluated. Volume of each interior (light coloured) pie-chart slice represents the %SNP for the functional annotation, which is equal to the expected 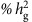 under the null of no enrichment. Volume of each shaded pie-chart slice represents the actual 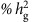 inferred by the model. Slices extending outside/inside the middle pie correspond to enrichment/depletion in SNP heritability, as indicated by the dotted lines. Colour coding is consistent across all subpanels. * (**) denotes significant deviation at *P*<0.05 (*P*<0.05/15) of fraction of SNP heritability (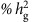 from null model of 
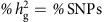
 by *Z*-test; see Supplementary Table 6 for *P* values).

Although the results above showcase the power of the variance component approach in finding epigenetic marks relevant for PrCa, such marks often overlap making the causal mark difficult to identify (Supplementary Fig. 1). To account for the correlation among marks we grouped the 82 marginally significant annotations into 15 biologically relevant, non-overlapping groups organized by mark and cell line, and partitioned 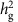 across all groups in a joint model (see Methods, Table 1, Fig. 5 and Supplementary Table 6). Five components were nominally significant in the joint model at *P*<0.05; out of the five components three remained significant after accounting for 15 tests: H3k27ac marks in LNCaP (*P*=2.5 × 10^−20^ by *Z*-test); DHS marks in other cancer cell types (*P*=3.9 × 10^−5^ by *Z*-test); and repressed segmentations (*P*=2.1 × 10^−20^ by *Z*-test). To further refine our model, we restricted to the significant annotations (and the background components accounting for LD to coding regions) and re-evaluated them jointly, referred to as the ‘selected' model. This selected model localized 51.0% of the 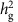 within 12.1% of SNPs (LNCaP: H3K27ac+ARBS+DHS cancer), whereas coding regions only explained 3.3% (s.e. 1.4%) of 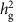 within 1.8% of SNPs (Supplementary Table 7). The localization was even stronger with imputed data, where 86% of the 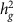 was localized to 8.6% of SNPs (Table 1 and Supplementary Tables 8 and 9). Estimates from imputed markers were more representative of underlying enrichment in our simulations (see Methods, Supplementary Table 2) but may include the effects of nearby markers^12^ and so we consider them as an upper bound. None of the estimates changed significantly after adjusting for known GWAS associations^2^ (79 of which were typed in this data), underscoring the polygenic nature of this effect.

Having inferred the selected model, we re-analysed each of the 82 marginally significant categories jointly with the selected model (see Methods). Only three marks remained significant: two H3k27ac annotations in the colon crypt and one H3k27ac annotation in pancreas (Supplementary Data). This implies that the marginal enrichment of the 82 annotations was primarily driven by the overlap with functional marks in the selected model. For example, the H3K4me1 mark in penis foreskin keratinocytes that was previously highly significant (24.6% 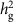, *P*=3.0 × 10^−16^ by *Z*-test, Fig. 1) was no longer enriched after conditioning on the selected model (7.1% 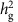, *P*=0.29 by *Z*-test, Supplementary Data). The reduction to a small number of categories in the selected model with limited loss in signal further emphasizes the extent to which the selected model has localized the functional sources of enrichment. Focusing on the two most enriched categories in the selected model, we found that SNPs present in both the prostate tissue ARBS and LNCaP H3k27ac marks yielded significantly higher average heritability per SNP than either mark individually (Supplementary Table 10). In contrast, the variants specific to ARBS or H3k27ac were comparable in SNP heritability.

### Replication of genomic functional atlas across ancestries

We evaluated replication of our model using two separate genome-wide SNP data sets of PrCa, one of European ancestry (BPC3; 6,953 samples) and one of African ancestry (AAPC; 9,522 samples) for PrCa (see Methods). To account for the smaller sample size, we focused on the eight-component selected model, only retaining significant components and three coding-proximal classes (coding, UTR, promoter)^12^. Because of platform differences between the populations, we used post-QC imputed variants in each data set, which are most reflective of underlying enrichment in our simulations (see Methods). We replicated the significant deviation in 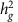 at H3k27ac and the repressed loci across both BPC3 and AAPC (Supplementary Tables 11 and 12). However, cancer DHS was only significant in the BPC3 data and ARBS not significant in either (though the estimates were not significantly different from the iCOGS estimate). The enrichment did not change after restricting to very high-quality imputed markers (Supplementary Table 13). Although the relatively small validation sample size did not provide enough power to test differences between the ancestries, the mean SNP heritability for variants within each mark were remarkably similar (*ρ*=0.90 between AAPC and BPC3 across eight components), suggesting a similar pattern of aggregate contribution to risk coming from common variants marked by epigenetic classes across European and African American ancestries (though individual risk variants themselves may differ).

### H3k27ac mark in LNCaP is specific to PrCa

As a negative control, we evaluated the selected model with imputed SNPs across 11 common non-cancer diseases from the Wellcome Trust Case Control Consortium (WTCCC) (see Methods, Supplementary Table 14) where we observed two main differences: the LNCaP H3k27ac annotation was no longer significantly enriched (1.1% 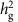 with 2.6% of SNPs); and the repressed regions were much less depleted from the null (28.1% 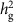 with 87.8% of SNPs) compared with the 0.3% of 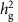 observed in iCOGS imputed data (*P*=2.2 × 10^−4^ for difference by *Z*-test). Interestingly, although ARBS were significantly enriched in all 11 traits, the enrichment was no longer significant after excluding autoimmune traits. Overall, these differences indicate that the LNCaP H3k27ac mark is uniquely informative for PrCa, whereas variants near the ARBS and DHS cancer elements (which overlap other DHS annotations by 56%; Supplementary Fig. 2) may play a generally important role across other common diseases^12^.

### Genomic functional atlas improves polygenic risk prediction

To validate our SNP heritability genomic atlas, we compared the accuracy of predicting case/control status from genetic data with or without the functional atlas. We evaluated three distinct prediction models in the iCOGS sample: (i) a genetic risk score (GRS) from the genome-wide significant SNPs; (ii) the single best linear unbiased predictor (BLUP) using a single variance component from all SNPs; and (iii) the weighted sum of individual BLUPs from each epigenetic category in the selected model (multi-BLUP; see Methods). Evaluated by cross-validation, the GRS yielded an *R*^2^=0.029 with true phenotype, whereas the single BLUP yielded an *R*^2^=0.065 and the multi-BLUP had an *R*^2^=0.071 (Supplementary Table 15). In a joint model with all three predictors, the multi-BLUP was highly significant (*P*=5.3 × 10^−31^ from multiple regression). When we constructed the GRS from SNPs recently discovered in a much larger PrCa GWAS (ref. 2), the resulting prediction *R*^2^ increased to 0.084. However, including the single BLUP or the multi-BLUP as an additional predictor still increased the prediction *R*^2^ to 0.096 (joint *P*=6.7 × 10^−4^ from multiple regression) and 0.098 (joint *P*=1.3 × 10^−23^ from multiple regression), respectively (Supplementary Table 15). The consistent statistical significance and increased prediction accuracy confirms the validity of the selected model in this data and in larger GWAS.

## Discussion

Using large-scale genotype data from over 59,089 men of European and African American ancestries jointly with epigenetic annotations, we identified highly significant differences in SNP heritability 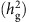 of PrCa across variants from different epigenetic classes, tissue types and cell lines. Focusing on marks measured in prostate, we observed significantly higher 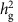 around tumour-specific ARBS; ARBS measured in primary tissue relative to cell line; and DHS measured in PrCa cell line relative to prostate epithelial cell line. The enrichment at tumour-specific ARBS was consistent with recent findings showing that these sites were enriched for nearby genes highly expressed in tumours^18^. These analyses are comprehensive and cover most commonly studied prostate cell lines except for vertebral cancer of the prostate, which were not well represented in the ENCODE/ROADMAP. A search across 544 diverse functional annotations restricted most of the 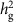 to a small fraction of the genome marked by prostate regulatory elements. Consistent with previous findings in common disease, functionally repressed regions were significantly depleted in heritability, highlighting the role of active regulation in PrCa susceptibility. Subsequent model selection localized the enrichment from 82 individually significant annotations to six that remained significant in a joint model. In particular, the abundance of enrichment in H3k27ac marks (active enhancers) relative to H3k4me1/H3k4me3 (poised enhancers/promoters) underscores their role in PrCa, though further enrichment in super enhancers was not observed. The enrichment within LNCaP: H3K27ac and depletion at repressed regions was replicated across different ancestries and yielded significant improvements in polygenic risk prediction.

With most GWAS associations falling outside coding regions, our analyses offer an important resource for prioritizing potential loci and focusing future studies on the most heritable genomic regions^27^. The marginal analyses provide a ranking of 544 common functional assays, while the selected model localizes heritability to only those functional classes that are independently enriched. Emerging functional categories may further refine this signal or reveal other relevant epigenetic marks, though little enrichment beyond the selected model was observed in the comprehensive sampling of functional data analysed here. In general, the variance component model offers an opportunity to evaluate biological hypotheses *in silico* and without strictly relying on individually significant SNPs. However, as with any analysis of array-based data, the 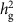 estimates will not include the contribution of SNPs that are untyped or poorly tagged, such as rare variants or other contributors to the missing heritability. Future analyses of whole-genome sequencing, additional functional annotations, and larger sample sizes can yield important insights into functional mechanisms that are still not localized. Overall, our results suggest similar patterns of functional enrichment across men of European and African American ancestry and highlight ARBS, H3k27ac marks in LNCaP cell lines and DHS in cancer cell lines for follow-up studies of PrCa risk.

## Methods

### Epigenetic annotations

Sample collection and processing for functional annotations was made publically available by the ENCODE/ROADMAP consortia^28^. DHS, H3k4me1, H3k4me3, H3k9ac annotations and genome segmentations^20^,^29^, enhancers and super enhancers^26^ and PrCa-specific annotations^7^,^18^ were assay and processed as detailed in the original studies. Tumour-only and normal-only ARBS were defined in seven normal and 13 tumour specimens in the original study^18^. All annotations curated for this paper (ENCODE/ROADMAP; Pomerantz *et al*.; and Hazelett *et al*.) are available at https://data.broadinstitute.org/alkesgroup/ANNOTATIONS/PRCA/. The full list of individual annotations with web-links to the corresponding boundary definitions is provided in the Supplementary Data. Some functional marks are listed multiple times due to multiple independent assays or laboratory protocols.

### ARBS ChIP-seq in human tissue specimens

The ARBS assay was performed as described in REF (ref. 18) and summarized here. Fourteen subjects of European American ancestry were selected for ChIP analysis. Their chromatin was incubated overnight with 6 μg antibody AR (N-20, Santa Cruz Biotechnology, Dallas, TX) bound to protein A and protein G beads (Life Technologies, Carlsbad, CA). A fraction of the sample was not exposed to antibody to be used as control (input). The samples were de-crosslinked, treated with RNase and proteinase K, and DNA was extracted. The samples were then re-sheared to 100–300 base pairs using the Covaris ultra-sonicator, and concentrations of the ChIP DNA were quantified by Qubit Fluorometer (Life Technologies). DNA sequencing libraries were prepared using the ThruPLEX-FD Prep Kit (Rubicon Genomics, Ann Arbor, MI). Libraries were sequenced using 50-base pair reads on the Illumina platform (Illumina, San Diego, CA) at Dana-Farber Cancer Institute. AR binding sites were generated using Model-Based Analysis of ChIP-seq 2 (MACS2), with a qvalue (false discovery rate, FDR) threshold of 0.01.

The 13 tumours used in this study were androgen dependent and not exposed to androgen deprivation therapies. All of the tumours were specimens obtained from radical prostatectomies, derived from men with early stage disease. These samples were not selected based on any specific features; therefore, we would expect that the distribution of risk variants would be similar to a random sampling of PrCa cases. Large-scale genetic surveys have shown that somatically acquired alterations in primary localized prostate tumours (the type of tumour evaluated in this study) are infrequent. Based on these previous results, we believe that somatically acquired genetic events in regions related to androgen biology are not common and, therefore, do not influence our results.

### Patient material

Informed consent was obtained from all subjects and all studies were approved by local Research Ethics Committees and/or Institutional Review Boards.

### Data quality control

Quality control is crucial for accurate heritability estimation, where many small artifacts can add up to large biases. All data sets went through a stringent QC process with the following exclusion criteria: minor allele frequency (MAF)<1%; fraction of missing/uncalled SNPs>5%; Hardy–Weinberg equilibrium *P* value<0.01; case–control missingness *P* value<0.05; imputation INFO score>0.30. In addition, close relatives were pruned such that no pair of individuals had genetic relatedness (GRM) coefficients>0.05. The top 10 principal components and a coded study label were always included as fixed-effects. All analysed samples, cases and controls, were males.

### iCOGS data

The iCOGS consortium genotyped balanced cases and controls on a custom targeted array^4^. After quality control, 42,613 samples and 153,621 genotyped SNPs remained. Imputation was performed to the 1000 Genomes reference panel using HAPI-UR (ref. 30) for phasing and IMPUTE2 (ref. 31) for imputation. Overall, 1,910,827 imputed and genotyped SNPs passed QC. Because of computational restrictions, the heritability estimation was carried out in two equally sized halves of the ICOGS, with total effects computed by inverse-variance meta-analysis. We partitioned the genotyped SNP heritability by MAF but observed no trend and only slight enrichment of % 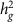 at high-frequency variants (Supplementary Table 16).

### BPC3 data

The National Cancer Institute Breast & Prostate Cancer Cohort Consortium (BPC3) consortium genotyped individuals on the Illumina HumanHap610 quad array^32^. After quality control, 6,953 samples and 4,004,229 genotyped and imputed SNPs remained. Age was available for all samples and additionally included as a covariate.

### AAPC data

The AAPC consortium genotyped individuals of African ancestry on the Illumina Human1M array^2^,^33^,^34^. After quality control, 9,522 samples and 10,468,389 genotyped and imputed SNPs remained.

### WTCCC data

The Wellcome Trust Case Control Consortium Genotyping genotyped cases for 11 traits as well as shared controls on multiple Illumina and Affymetrix arrays^35^,^36^,^37^. The phenotypes analysed here were ankylosing spondylitis (AS); bipolar disorder (BD); coronary artery disease (CAD); Crohn's disease (CD); hypertension (HT); multiple sclerosis (MS); rheumatoid arthritis (RA); schizophrenia (SP); type 1 diabetes (T1D); type 2 diabetes (T2D); and ulcerative colitis (UC). After quality control, a total of 47,053 samples and 4–5 million genotyped and imputed SNPs remained. Reported 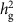 values were estimated for each phenotype separately and meta-analysed using inverse-variance weighting.

### UK10K data

The UK10K whole-genome sequence data from ALSPAC and TWINSUK (http://www.uk10k.org) was used only for simulation, and so stringent quality control was not applied. After relatedness filtering, 3,047 samples and 15,691,225 non-singleton variants were retained.

### Heritability estimation of individual annotations

We estimated the SNP heritability 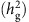 captured by functional categories in a joint variance component model using GCTA as described in REF (ref. 20). Briefly, this model assumes the phenotype is drawn from a multivariate normal distribution with variance-covariance modelled by components computed from the SNPs and a normal residual. For each functional category (for example, DHS) *i*=1..*M* where *M* is the total number of categories in the model, a GRM across all pairs of individuals is computed restricting to SNPs within the functional category. Variance components for all GRMs in the model are then fitted using REML as implemented in GCTA to estimate a variance parameter 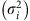 used to compute 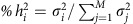. The 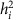 corresponds to the fraction of trait variance that can be explained by the BLUP restricted to SNPs in the corresponding functional category (or annotation). For a given functional annotation, SNPs were categorized into a hierarchy of seven non-overlapping components: (1) coding; (2) UTR; (3) promoter (functional annotation of interest); (4) DHS; (5) intron; and (6) intergenic. SNPs belonging to multiple categories were partitioned explicitly into the first category in this list. The coding and coding-proximal components were included to ensure that the annotation heritability was not inflated by SNPs that were in high LD with coding variation. A genetic relatedness matrix was computed for each component by first standardizing the corresponding SNPs and then computing a SNP covariance over all pairs of samples. Component-specific *σ*^2^ and errors were fitted iteratively using the Average Information algorithm^38^. The analytical standard error for 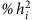 was estimated by transforming the GCTA-inferred 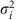 and error covariance matrix using the delta method. As in REF (ref. 20) statistical significance was evaluated by comparing the 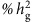 explained by the category and it's standard error to the %SNPs in the category using a *Z*-test (comparing nested models using a likelihood ratio test yielded similar results). Total 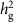 estimates were computed as 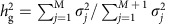 after transforming to the liability scale assuming a prevalence of 0.14 and using the study-specific case/control ratio.

### Hierarchical joint models

For specific models of interest, we extended the individual annotation model described above to test intersecting and non-intersecting components. This allowed us to evaluate precisely which sub-annotations of overlapping components were likely to be causal. For the tumour/normal model, we expanded each tumour/normal mark by 5 kb in both directions from the center to capture nearby genes and other regulatory regions so that tumour (normal) covered 3.3% (1.4%) of the SNPs, respectively. We estimated 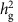 from the joint hierarchical model: (1) coding; (2) UTR; (3) promoter; (4) normal-only; (5) tumour-only; (6) DHS; (7) intron; and (8) Other. When comparing ARBS from tissue and ARBS LNCaP from cell line, only 59 SNPs (0.03%) overlapped between the two categories, and so we tested two separate models: (1) coding; (2) UTR; (3) promoter; (4) (ARBS tissue/ARBS LNCaP); (5) DHS; (6) intron; and (7) other. For comparisons between LNCAP, PREC and RWPE1 using DHS we tested each pair of cell lines using the joint model: (1) coding; (2) UTR; (3) promoter; (4) DHS particular to one cell line; (5) DHS common to both cell lines; (6) DHS particular to other cell line; (7) DHS other cell lines; (8) Intron; and (9) Other. For comparisons between enhancers and super enhancers, we used the 86 cell-type-specific annotations from REF (ref. 26), testing each enhancer or super enhancer separately in the following joint model: (1) coding; (2) UTR; (3) promoter, (4) (enhancer/super enhancer for cell-type of interest); (5) DHS; (6) intron; (7) other. Of these, 49 cell types yielded model convergence for both the enhancer and corresponding super enhancer and were used to estimate means and correlation. The order and grouping of marginally significant annotations into epigenetic mark and cell type (for example, in Table 1) are listed in the Supplementary Data. For each of the 82 individually significant annotations, we re-evaluated them jointly with the selected model in the following hierarchical joint model: (1) coding; (2) UTR; (3) promoter; (4) LNCaP:H3k27ac; (5) ARBS; (6) DHS cancer; (7) (functional annotation of interest); (8) DHS; (9) intron; and (10) other. Only functional annotations that converged were reported in the Supplementary Data.

### Accuracy of 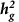 estimates from typed variants in simulations

The variance component model has previously been shown to yield robust estimates under the assumption that causal variants are typed and uniformly sampled from a given functional category^13^,^20^,^21^. Here, we perform simulations using the UK10K whole-genome sequence data to confirm the validity of this model for our annotations, and to assess how representative SNP estimates are of true underlying biology at common sequenced variants. Overall, the simulations involve using real markers to generate additive, polygenic phenotypes with a given heritability and then estimating the heritability with the variance component model. We evaluated the UK10K data for three types of SNPs: (i) common sequenced variants (7,534,538 SNPs); (ii) UK10K SNPs typed by the iCOGS platform (178,509; 95% of iCOGS SNPs); and (iii) UK10K SNPs typed and imputed by the iCOGS platform (1,655,723; 87% of the iCOGS imputed SNPs). We focused on the LNCaP:H3k27ac annotation (which was most significant in our data) to evaluate the main joint model. All phenotypes were simulated by drawing 5,000 causal variants randomly from the specified categories and sampling causal effect-sizes from a normal distribution such that SNPs either explain equal variance (the model assumption) or variance in proportion to their MAF. The phenotype was then generated as the dot product of genotype and effect-size with random noise added to fix heritability at 50%. Phenotypes were simulated thousands of times until the standard error over simulations was low enough to evaluate unbiasedness.

We confirmed that estimates of 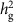 from a polygenic trait were accurate under the model where causal variants are typed (Supplementary Table S1). Under the null, the LNCaP H3k27ac component is expected to explain 3.22% of the SNP heritability, and the model estimated 3.50% (0.22%) and 3.68% (0.21%) under a low-frequency and high-frequency disease architecture, respectively (Supplementary Table S1). None of the estimates were significantly different from the truth given the number of components tested. Under a scenario where LNCaP H3k27ac explains 50% of the 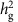, the model estimated 51.13% (0.40%) and 46.98% (0.35%) under a low-frequency and high-frequency disease architecture, respectively (Supplementary Table S1). Although the high-frequency architecture (where common variants explain more variance in trait than rare variants) represents a substantial model misspecification, our simulations show that this does not introduce substantial bias and is likely to slightly underestimate the SNP heritability at the focal chromatin mark. In all cases, the empirical standard deviation over 500 simulations was similar to the average analytical s.e.m. computed by GCTA (REML algorithm), thus showing that that analytical standard error is well calibrated (Supplementary Table S1). We note that the standard error is inversely related to the sample size^39^,^40^, and is therefore much higher in these simulations than in the iCOGS data which is 14-fold larger.

Lastly, we performed the real data partitioned analysis in subsets of individuals to evaluate biasedness and power to detect significant enrichment. We confirmed that no significant differences were observed between estimates from the entire study compared with those averaged across subsets of the study (Supplementary Fig. 3). As such, we can confidently report estimates and bounds on the enrichment observed in the entire study that will hold for larger studies. Furthermore, all but one of the significant components from the main model remained significant in smaller samples (ARBS), making it unlikely that they were affected by winner's curse. Recent work has quantified the theoretical relationship between estimation error and effective sample size for individual components^39^,^40^.

### Causal variants not tagged on the iCOGS genotyping platform

We used the sequenced UK10K common variants to evaluate how well the iCOGS genotyped and imputed SNPs captured underlying heritability by simulating phenotypes using causal variants from sequencing and estimating heritability from the iCOGS SNPs (that is, hiding variants that were not genotyped or imputed, Supplementary Table S2). 83% of common UK10K SNPs lie within 100 kb of an iCOGS SNP, so some common variation is likely to be partially tagged by the chip. If the imputed and/or genotyped SNPs served as a good proxy for the common sequence variation, then we would expect their estimates of 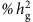 to match the simulated fractions. When no functional category was enriched with causal variants, small but significant differences were observed for genotyped coding variants (4.75% 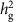 estimated as compared with simulated 0.67%) and imputed intergenic variants (56.09% 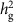 as compared with 50.52% simulated) but not the focal LNCaP:H3k27ac category. Similar deviations were observed for the disease architecture where common variants explain more variance in trait than rare variants (Supplementary Table S2). When causal variants where enriched within LNCaP:H3k27ac category, deviations between simulated and estimated SNP heritability were larger (Supplementary Table S2). Most of this deviation was due to a significant underestimate at LNCaP:H3k27ac, which was simulated to explain 50% of 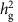 but explained only 12.55% (s.e.m.0.92%) and 30.92% (s.e.m. 1.09%) from genotyped and imputed SNPs, respectively. This heritability was distributed across all the remaining components, particularly in intergenic SNPs for the genotyped estimate and DHS SNPs for the imputed estimate, which tend to be nearby.

Overall, our simulations showed that the model is highly accurate when all causal variants are typed. When considering enrichment from untyped causal variants, the imputed estimate was consistently closer to the truth than the genotyped estimate. Most importantly, the estimate from the focal category (LNCaP H3k27ac in our simulations) was shown to be highly conservative both in the null and in the enriched scenario and unlikely to be biased due to tagging of untyped markers. We note that previous work has shown estimates from imputed SNPs (but not genotyped SNPs) may be contaminated by markers very close to an enriched annotation^12^; as such we focused our results on the densely genotyped iCOGS variants which are expected to be conservative, and primarily used imputed data for validation across data sets.

### Estimates of 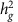 from African American samples

To assess potential biases in estimating 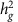 from an admixed population, we performed separate simulations in the AAPC data where causal variants were specifically sampled from varying *F*_ST_ bins. This framework evaluated the potential bias resulting from markers that had drifted to different frequencies in the two populations. The *F*_ST_ was estimated out-of-sample in the HapMap CEU European and YRI Yoruba populations. We tested the null six-component model (Coding, UTR, Promoter, DHS, Intron, Other) and observed no significant deviations from the null under any class of differentiated SNPs (Supplementary Table S3). However, we note that total 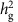 was simulated at 0.50 but was inferred at 0.38–0.66 across increasing quintiles of causal *F*_ST_ (Supplementary Table 3), indicating that even with well-calibrated estimates of enrichment the total estimate may be biased upwards if the causal SNPs are highly differentiated (observed in this simulation when mean causal *F*_ST_>=0.35).

### Genetic prediction

We sought to validate the utility of our functional atlas by applying it to genetic prediction. The aim of genetic prediction is to use training individuals with genetics (for example, SNPs) and diagnosed phenotype to accurately predict the phenotype into individuals with only genetic data available^41^,^42^. Here, we focus on correlation of predicted phenotype with true phenotype (*R*^2^), as it has a natural relationship to SNP heritability^12^,^42^. Intuitively, better localization of the true effect-sizes will reduce noise in training the predictor and increase accuracy. If the functional atlas identified regions with increased heritability, this information should significantly improve the prediction. We evaluated three standard models of risk prediction: GRS; BLUP (ref. 43); and multi-component BLUP (ref. 14). The GRS was computed as a sum over SNPs of the log odds-ratios from the training sample^41^. The set of SNPs used was either the genome-wide significant markers in the training set (restricted to one per 1 MB locus) or the genome-wide significant markers identified in a recent large GWAS of PrCa^2^. In contrast to the GRS, the BLUP used all markers in the data to form the prediction. The standard BLUP was estimated using GCTA over all SNPs. The multi-component BLUP was estimated using the components in the selected model (jointly) to compute a single score equal to the sum of the predictions from each component weighted by their component-specific 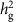. This is analogous to specifying a different prior on the effect-size variance in each component. All predictions were carried out by cross-validation in the full iCOGS data, removing 1,000 individuals in each fold. Prediction R^2^ was then computed from a regression of phenotype on the predictor score with 10 PCs included as covariates to account for ancestry, subsequently subtracting the *R*^2^=0.021 from a model with PCs only. *P* values were estimated for each of the coefficients in the multiple regression of phenotype ∼ GRS+single-BLUP+multi-BLUP+PCs. To ensure that prediction across data sets was independent, we carefully removed all iCOGS individuals with a GRM value of >0.05 to any individual in the BPC3 when computing BLUP coefficients. We separately analysed the predictor in 26,000 iCOGS samples that had age at diagnosis, but did not observe significant differences before/after including age as a covariate.

## Additional information

**How to cite this article:** Gusev, A. *et al*. Atlas of prostate cancer heritability in European and African-American men pinpoints tissue-specific regulation. *Nat. Commun.* 7:10979 doi: 10.1038/ncomms10979 (2016).

## Supplementary Material

Supplementary Information Supplementary Figures 1-3, Supplementary Tables 1-16 and Supplementary Note 1

Supplementary Data 1 Estimates of partitioned heritability from all analyzed annotations, with corresponding web resource

## Figures and Tables

**Table 1:** Partitioning of heritability across functional classes in prostate cancer.

**Functional category**	**%SNPs**	**Full Model**		**Selected model**					
		**iCOGS genotyped**		**iCOGS imputed**		**BPC3 imputed**		**AAPC imputed**	
		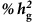	**s.e.m.**	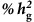	**s.e.m.**	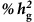	**s.e.m.**	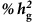	**s.e.m.**
Coding	1.8	3.0	1.3	0.9	2.9	0.2	10.1	3.3	11.1
UTR	1.9	1.6	1.4	3.0	3.1	21.0	11.3	5.9	11.2
Promoter	3.4	*7.8	1.8	8.9	4.1	0.0	12.7	0.0	14.7
LNCaP: H3k27ac	3.2	**22.3	2.1	**27.0	3.8	*30.3	12.1	*28.9	12.7
ARBS	1.0	*3.3	1.1	*9.1	3.3	1.1	12.1	15.2	12.1
LNCaP: FOXA1	1.5	1.5	1.3						
LNCaP: H3k4me1	2.0	1.3	1.4						
LNCaP: DHS	2.9	5.4	1.6						
DHS prostate	1.8	2.6	1.4						
DHS cancer	4.7	**14.1	2.3	**49.6	6.3	*47.4	21.4	46.6	22.4
H3k4me1 (other)	16.3	19.6	3.5						
H3k27ac (other)	7.3	4.1	2.4						
DHS (other)	1.8	0.2	1.3						
repressed	48.7	**11.0	4.1	**0.3	7.0	**0.0	23.8	**0.0	24.5
all other	1.7	0.7	1.2	0.2	2.7	0.0	9.2	0.0	7.6

ARBS, androgen receptor-binding sites; DHS, DNase I hypersensitivity sites; SNP, single-nucleotide polymorphism; UTR, untranslated region.

Full model denotes a 15-variance components model while ‘selected' model denotes a model restricted to the five components attaining significance in the ‘full' model (and three components for background). * (**) denotes significant deviation at *P*<0.05 (*P*<0.05/15) of fraction of SNP heritability 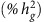 from null model of 
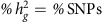
 (by *Z*-test; see Supplementary Table 6 for *P* values).
