# Supplementary material for: Atlas of prostate cancer heritability in European and African-American men pinpoints tissue-specific regulation
Source: Nat Commun. 2016 Apr 7;7:10979. doi: 10.1038/ncomms10979 (PMC4829663; doi:10.1038/ncomms10979)
Supplement: Supplementary Information — Supplementary Figures 1-3, Supplementary Tables 1-16 and Supplementary Note 1 [file ncomms10979-s1.pdf]

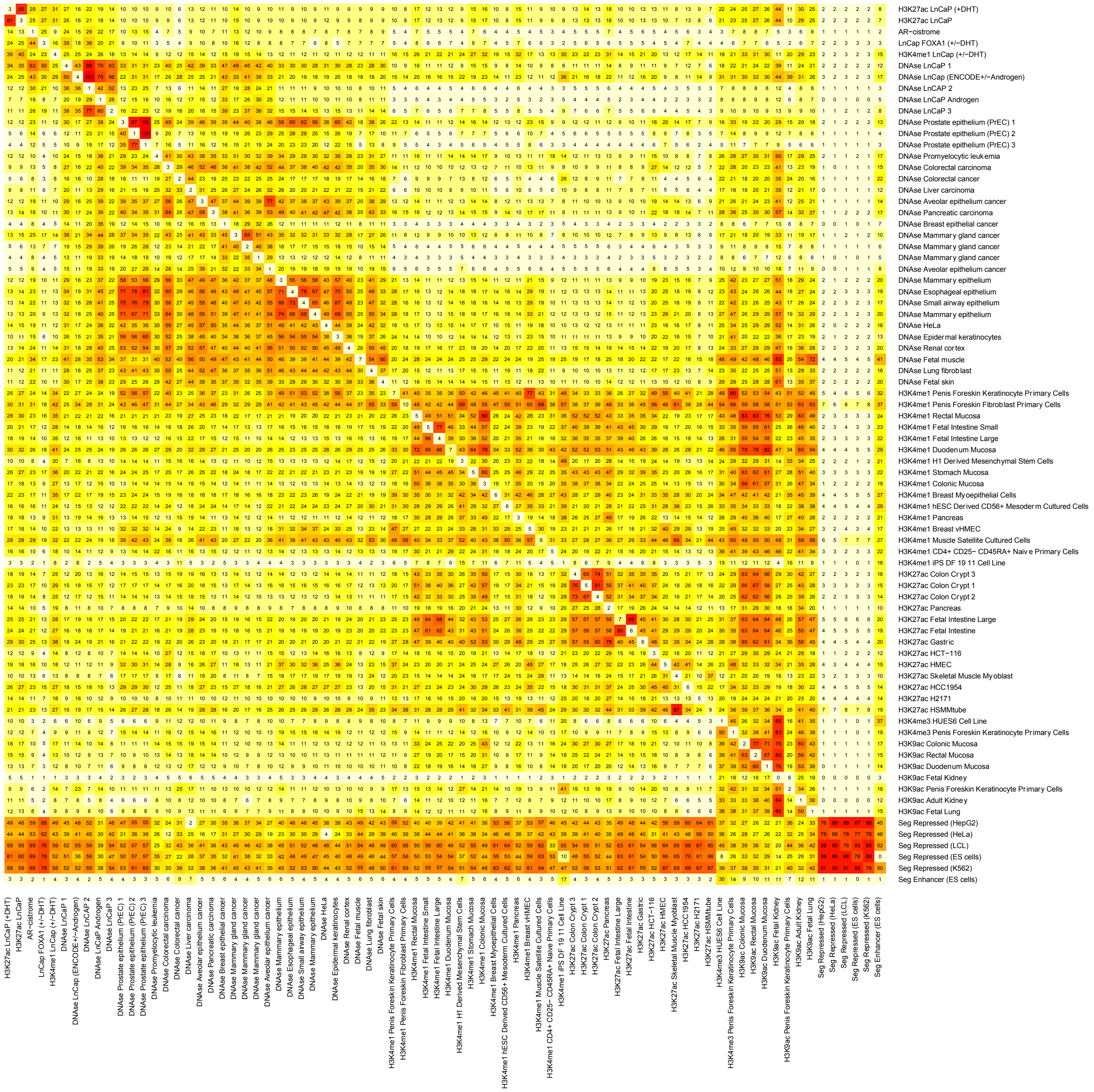

Supplementary Figure 1: SNP overlap of significant annotations in ICOGS. The percentage of SNPs in column-annotation that overlap SNPs in row-annotation are reported in each cell. The diagonal lists the fraction of total SNPs in that annotation.

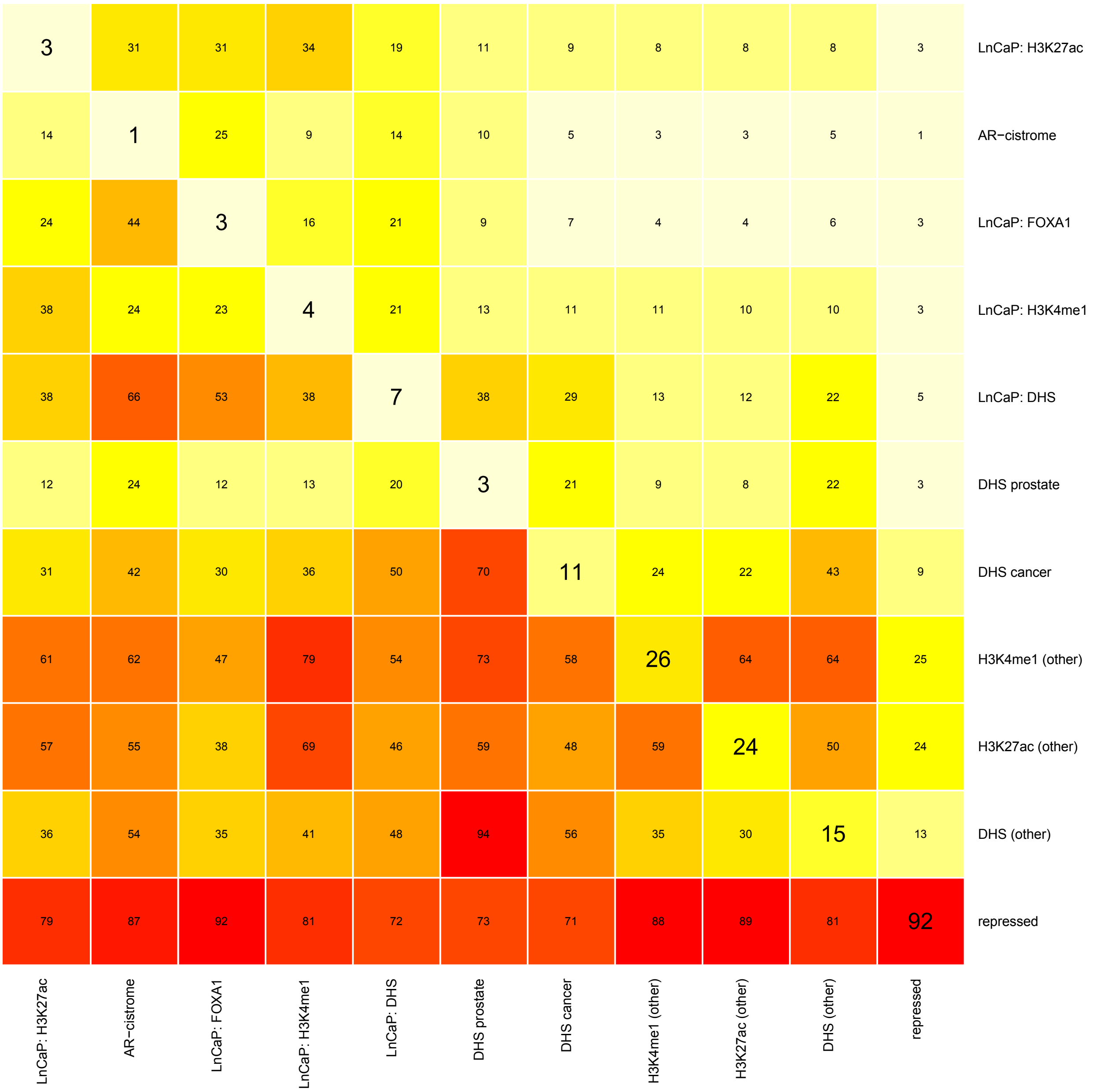

Supplementary Figure 2: SNP overlap of selected model annotations in ICOGS. The percentage of SNPs in column-annotation that overlap SNPs in row-annotation are reported in each cell. The diagonal lists the fraction of total SNPs in that annotation.

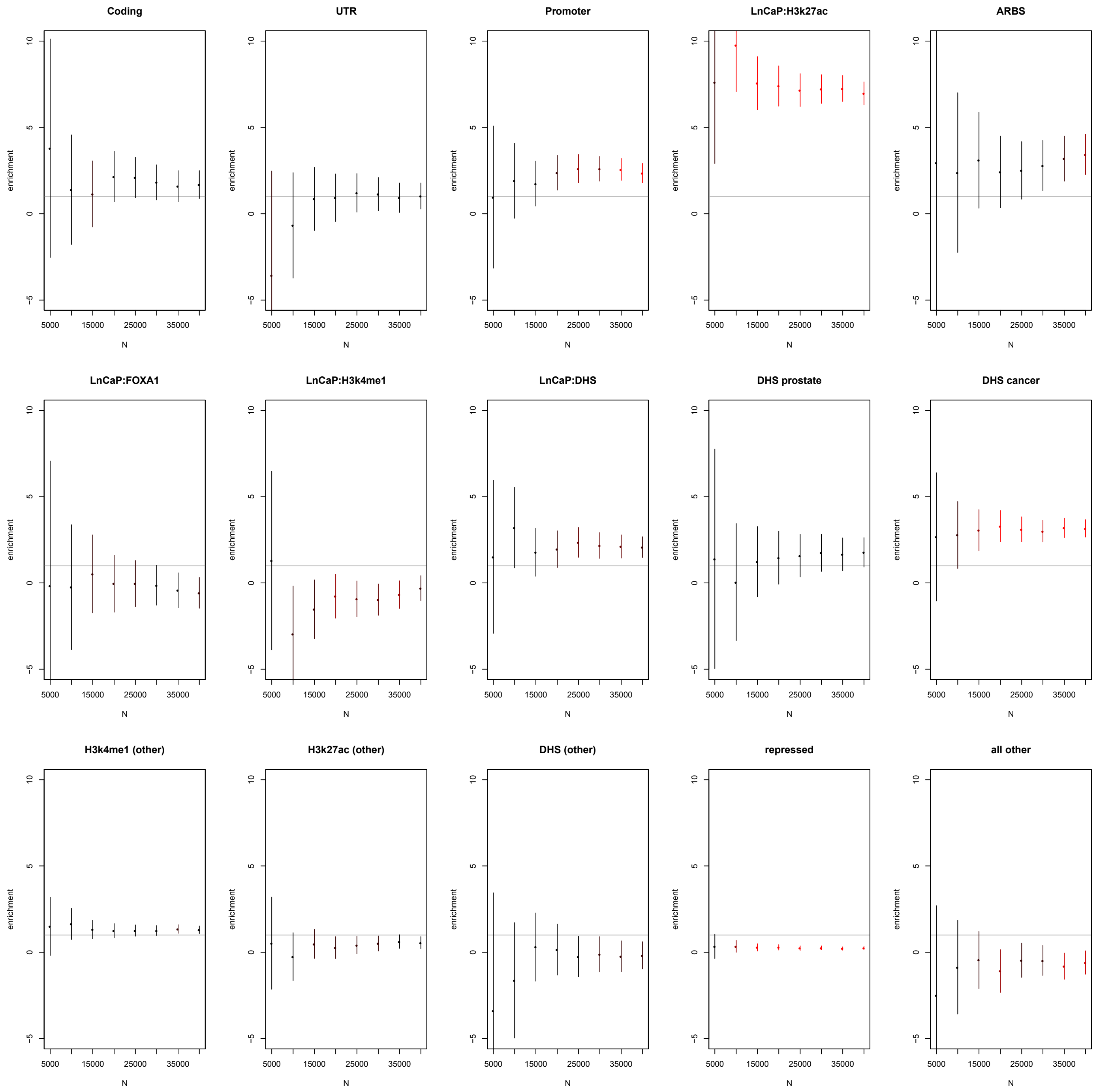

Supplementary Figure 3: Estimated enrichment from smaller sample sizes. The iCOGS cohort was randomly sub-sampled and heritability enrichment re-estimated for each component in the joint model from a subgroup. Each subfigure shows the estimated enrichment and standard error for the named component as a function of sample size, with each point corresponding to five random samples. The point color corresponds to the fraction of subsamples that were significantly enriched/depleted. Estimates at lower sizes are unbiased with respect to the full sample.

Supplementary Table 1: Simulations using UK10K sequencing data over 3,047 individuals show that variance components estimation attains high accuracy. To assess potential biases in variance components estimation, we simulated phenotypes starting from the real UK10K sequencing data and employed the variance components framework to estimate heritability attributable to each category of variants. First we sampled 5,000 SNPs from those on the iCOGS genotyping platform to serve as causal variants; second, we sampled effect sizes either inversely proportional to SNP variance (each SNP explains equal variance in trait, in expectation) or from the standard normal (high frequency SNPs explain more variance in trait than low frequency SNPs); and third, simulated additive polygenic phenotypes for each individual. We report the GCTA inferred $\%h_g^2$ from simulations where causal effects within LNCaP: H3k27ac are not enriched (top) or enriched to explain 50% of the SNP-heritability (bottom). The standard deviation ("s.d." column) across simulations was similar to the analytical standard error computed by GCTA (REML algorithm) ("AI s.e." column) thus showing that GCTA's analytical standard error is well calibrated. The SNP-heritability estimated by REML was very similar to the true simulated SNP-heritability across all simulations. Overall, the simulations show that variance components approaches yield unbiased estimates and well-calibrated standard errors.

**LNCaP: H3k27ac category not enriched with causal variants**							
		All SNPs explain equal variance in trait			Common SNPs explain more variance		
**Category**	**True % h_g^2**	**Estimated % h_g^2 (s.e.)**	**s.d.[a]**	**AI s.e.[b]**	**Estimated % h_g^2 (s.e.)**	**s.d.[a]**	**AI s.e.[b]**
Coding	1.75%	1.47% (0.21%)	18%	21%	1.65% (0.19%)	14%	14%
UTR	1.88%	2.11% (0.23%)	18%	21%	1.05% (0.19%)	14%	14%
Promoter	3.44%	2.87% (0.27%)	22%	25%	4.09% (0.24%)	16%	17%
LNCaP: H3k27ac	3.22%	3.50% (0.22%)	17%	19%	3.68% (0.21%)	13%	13%
DHS	22.72%	22.62% (0.67%)	41%	48%	22.57% (0.63%)	38%	40%
Intron	26.97%	28.20% (0.53%)	34%	39%	27.90% (0.52%)	30%	31%
Other	40.04%	39.22% (0.57%)	36%	41%	39.06% (0.50%)	32%	33%
**LNCaP: H3k27ac category enriched with causal variants**							
		All SNPs explain equal variance in trait			Common SNPs explain more variance		
**Category**	**True % h_g^2**	**Estimated %h_g^2 (s.e.)**	**s.d.[a]**	**AI s.e.[b]**	**Estimated % h_g^2 (s.e.)**	**s.d.[a]**	**AI s.e.[b]**
Coding	0.88%	0.64% (0.18%)	19%	20%	1.02% (0.19%)	13%	14%
UTR	0.94%	0.56% (0.19%)	17%	20%	0.45% (0.18%)	17%	15%
Promoter	1.72%	3.02% (0.25%)	22%	23%	2.30% (0.24%)	18%	18%
LNCaP: H3k27ac	50.00%	51.13% (0.40%)	19%	22%	46.98% (0.35%)	18%	18%
DHS	11.36%	12.39% (0.64%)	43%	49%	12.19% (0.59%)	36%	40%
Intron	13.48%	12.78% (0.48%)	36%	39%	14.52% (0.45%)	34%	32%
Other	20.02%	19.47% (0.54%)	36%	41%	22.54% (0.49%)	33%	34%

[a]Empirical standard deviation across 500 simulations.
[b]Mean AI-REML estimate of standard error across 500 simulations (used for significance testing in real data).

Supplementary Table 2: Simulations using UK10K sequencing data over 3,047 individuals to assess the tagging properties of SNPs on the iCOGS genotyping platform. We simulated phenotypes starting from the real UK10K sequencing data and employed the variance components framework to estimate heritability attributable to each functional category using only the SNPs present on the iCOGS platform ("iCOGS" columns) or 1000 Genomes SNPs imputed from the iCOGS data ("Imputed" columns). We report the inferred $\%h_g^2$ from simulations where causal effects within LNCaP: H3k27ac are not enriched (top) or enriched to explain 50% of the SNP-heritability (bottom). In both scenarios, we observe that variance components using only iCOGS SNPs underestimate the $\%h_g^2$ coming from the LNCaP: H3k27ac category. However, using the imputed data attained an estimate closer to the simulated $\%h_g^2$ (e.g. 31% as opposed to 13% for the case when LNCaP: H3k27ac variants were simulated to explain 50% of all SNP-heritability). This suggests that our inferred $\%h_g^2$ results for LNCaP: H3k27ac using iCOGS SNPs are a lower bound on the total SNP-heritability attributable to LNCaP: H3k27ac in the iCOGS data (42,613 individuals, see main text).

**LNCaP: H3k27ac category not enriched with causal variants**					
		All SNPs explain equal variance in trait		Common SNPs explain more variance	
**Category**	**True % h_g^2**	**iCOGS % h_g^2 (s.e.)**	**Imputed % h_g^2 (s.e.)**	**iCOGS % h_g^2 (s.e.)**	**Imputed % h_g^2 (s.e.)**
Coding	0.67%	4.75% (0.84%)	0.18% (0.93%)	3.10% (0.68%)	-1.33% (0.82%)
UTR	0.89%	1.94% (0.80%)	1.95% (0.93%)	3.12% (0.69%)	2.04% (0.80%)
Promoter	2.62%	3.86% (1.04%)	5.17% (1.21%)	3.67% (0.81%)	6.77% (1.06%)
LNCaP: H3k27ac	2.50%	0.70% (0.82%)	0.14% (0.89%)	-0.19% (0.60%)	0.08% (0.73%)
DHS	15.10%	16.38% (2.14%)	15.21% (2.71%)	12.32% (1.80%)	15.32% (2.34%)
Intron	27.71%	22.28% (1.74%)	26.95% (1.82%)	28.30% (1.54%)	29.80% (1.68%)
Other	50.52%	49.72% (1.83%)	56.09% (1.92%)	50.90% (1.51%)	51.40% (1.57%)
**LNCaP: H3k27ac category enriched with causal variants**					
		All SNPs explain equal variance in trait		Common SNPs explain more variance	
**Category**	**True $\%h_g^2$**	**iCOGS % h_g^2 (s.e.)**	**Imputed % h_g^2 (s.e.)**	**iCOGS % h_g^2 (s.e.)**	**Imputed % h_g^2 (s.e.)**
Coding	0.34%	6.24% (0.82%)	0.91% (0.88%)	7.15% (0.61%)	0.98% (0.75%)
UTR	0.45%	6.53% (0.85%)	4.42% (0.85%)	5.83% (0.61%)	2.56% (0.73%)
Promoter	1.31%	4.58% (1.09%)	5.36% (1.09%)	3.22% (0.80%)	7.60% (1.02%)
LNCaP: H3k27ac	50.00%	12.55% (0.92%)	30.92% (1.09%)	13.45% (0.72%)	32.77% (0.94%)
DHS	7.55%	15.44% (2.09%)	16.16% (2.54%)	11.00% (1.64%)	12.90% (2.23%)
Intron	13.86%	21.80% (1.67%)	16.28% (1.74%)	22.80% (1.44%)	17.21% (1.46%)
Other	25.26%	37.67% (1.92%)	33.25% (1.70%)	38.50% (1.44%)	31.70% (1.52%)

Supplementary Table 3. Estimates of SNP-heritability from the African American samples. To assess potential biases in variance components estimation in admixed samples, we performed separate simulations in the AAPC data where causal variants were specifically sampled from varying fixation index (F_{ST}) bins. Effect sizes were sampled inversely proportional to the minor allele frequency in the study. This framework evaluated the potential bias resulting from markers that had drifted to different frequencies in the two populations. The F_{ST} was estimated out-of-sample in the HapMap CEU and YRI populations. First data row shows the total inferred h_g^2 (and standard error over all simulations), where the truth was simulated at 0.50; subsequent rows show the inferred $\%h_g^2$ for each functional category under simulations with no enrichment (category $\%h_g^2$ = category %SNPs). We tested the baseline six-component model (Coding, UTR, Promoter, DHS, Intron, Other) and observed no significant deviations from the null under any class of differentiated SNPs across 50 simulated phenotypes. The total estimate of h_g^2 was biased when unusually differentiated SNPs were causal due to different levels of LD at such SNPs, as has been shown previously (Speed et al. AJHG 2012).

		**1st F_{st} quintile**		**2nd F_{st} quintile**		**3rd F_{st} quintile**		**4th F_{st} quintile**		**5th F_{st} quintile**	
	**True h_g^2**	**h_g^2**	**s.e.**	**h_g^2**	**s.e.**	**h_g^2**	**s.e.**	**h_g^2**	**s.e.**	**h_g^2**	**s.e.**
Total	*0.50*	*0.38*	*0.01*	*0.38*	*0.01*	*0.43*	*0.01*	*0.50*	*0.01*	*0.66*	*0.01*
**Category**	**True % h_g^2**	**$\%h_g^2$**	**s.e.**	**$\%h_g^2$**	**s.e.**	**$\%h_g^2$**	**s.e.**	**$\%h_g^2$**	**s.e.**	**% h_g^2**	**s.e.**
Coding	1%	0.4%	0.8%	0.8%	0.8%	1.2%	0.6%	0.7%	0.6%	1.2%	0.4%
UTR	1%	0.4%	0.7%	0.6%	0.7%	2.1%	0.5%	1.4%	0.5%	1.1%	0.4%
Promoter	2%	2.8%	1.0%	2.6%	1.0%	2.0%	1.0%	2.5%	0.8%	2.7%	0.6%
DHS	23%	22.7%	2.4%	21.8%	3.0%	19.0%	2.8%	24.2%	2.7%	25.6%	1.5%
Intron	27%	29.9%	2.1%	27.1%	2.0%	28.4%	2.0%	26.8%	2.0%	27.2%	1.2%
Other	46%	43.8%	2.4%	47.1%	2.8%	47.3%	1.8%	44.4%	2.0%	42.3%	1.3%

Supplementary Table 4. Total SNP-heritability estimates and standard errors reported for the analyzed cohorts. BPC3 is the European genome-wide array study; AAPC is the African American genome-wide array study; and iCOGS is the custom chip used for main results (split into four equally sized sub groups for computational efficiency). BPC3 (h^2_{gwas}) corresponds to an estimate from 65 previously genome-wide significant SNPs in the BPC3. Bottom row reports the inverse-variance weighted meta-analysis estimates from the iCOGS. No significant heterogeneity was observed between the iCOGS sub-groups, and the iCOGS meta-analysis estimate does not differ significantly from the BPC3 or AAPC estimates. All estimates were converted to the liability scale using a prevalence of 0.14 and the listed case/control ratios.

Study	# Samples	Proportion of cases	h^2_g (liability scale)	h^2_g standard error (liability scale)
BPC3	6,953	0.39	0.26	0.05
BPC3 (h^2_{gwas})	6,953	0.39	0.06	0.001
AAPC	9,522	0.51	0.32	0.06
iCOGS (1/4)	10,990	0.52	0.29	0.03
iCOGS (2/4)	10.981	0.53	0.31	0.03
iCOGS (3/4)	10.354	0.39	0.25	0.03
iCOGS (4/4)	10.288	0.59	0.28	0.03
iCOGS meta	42,613		0.28	0.01

Supplementary Table 5. Functional annotations relevant to prostate cancer, corresponding heritability estimates, and significance of enrichment. #ID column corresponds to IDs in Supplementary Spreadsheet. REF indicates reference where the annotation was most recently evaluated.

#ID	type	cell line	Summary	REF	% SNPs	% h_g^2	$\%h_g^2$ standard error	p-value
458	DNAse	LNCaP (+/- AR)	prostate cancer (+/- androgen treatment)	ENCODE	4.2%	14.9%	1.6%	$2x10^{-11}$
511	DNAse	LNCaP (+ AR)	prostate cancer (+ androgen treatment)	ENCODE	0.8%	5.8%	0.8%	$4x10^{-09}$
459	DNAse	LNCaP	prostate cancer (UW FDR 0.05 peaks)	ENCODE	3.1%	16.7%	1.6%	$2x10^{-18}$
464	DNAse	LNCaP	prostate cancer (FDR 0.01 peaks)	ENCODE	1.5%	8.7%	1.1%	$6x10^{-11}$
537	DNAse	LNCaP	prostate cancer (FDR 0.01 Duke/UW combined peaks)	ENCODE	1.9%	8.4%	1.2%	$2x10^{-08}$
478	DNAse	PrEC	prostate epithelial (UW FDR 0.05 peaks)	ENCODE	2.8%	12.2%	1.4%	$4x10^{-11}$
479	DNAse	PrEC	prostate epithelial (FDR 0.01 peaks)	ENCODE	1.5%	7.5%	1.1%	$2x10^{-08}$
477	DNAse	PrEC	prostate epithelial (FDR 0.01 peaks)	ENCODE	1.2%	6.4%	1.0%	$1x10^{-07}$
481	DNAse	RWPE1	immortalized prostate epithelial cell-line	ENCODE	1.0%	2.2%	0.8%	$1x10^{-01}$
460	FOXA1	LNCaP	transcription factor FOXA1, facilitates androgen receptor binding	Hazelett	2.6%	9.4%	1.3%	$2x10^{-07}$
461	H3K27ac	LNCaP	histone H3 acetylated at lysine 27, distinguishes "active" enhancers	Hazelett	2.7%	20.4%	1.6%	$7x10^{-29}$
99	H3K27ac	LNCaP	histone H3 acetylated at lysine 27, distinguishes "active" enhancers	Hazelett	2.8%	13.6%	1.3%	$2x10^{-17}$
462	H3K27ac	LNCaP (+ DHT)	histone H3 acetylated at lysine 27, distinguishes "active" enhancers, treated with androgen dihydrotestosterone (DHT)	Hazelett	2.9%	22.2%	1.6%	$1x10^{-32}$
463	H3K4me1	LNCaP (+/- DHT)	histone H3 lysine 4 mono-methylation, distinguishes "poised" enhancers	ENCODE	3.7%	11.3%	1.4%	$2x10^{-07}$
8	ARBS	primary tissue	androgen receptor binding sites ascertained in 13 cancer and 6 normal samples (yielding 6.5x more sites than the ARBS LNCap below)	Pomerantz	1.5%	10.7%	1.2%	$1x10^{-14}$
543	ARBS	LNCaP (+ DHT)	androgen receptor binding sites	Hazelett	0.2%	3.2%	0.8%	$1x10^{-04}$
465	TCF7L2	LNCaP	transcription factor 7-like 2, abbarent expression implicated in cancer	Hazelett	0.4%	2.1%	0.8%	$4x10^{-02}$

Supplementary Table 6. SNP-heritability estimates using variance components in the full model using iCOGS imputed data over 42,613 individuals. We highlight in bold fonts the 3 functional annotations that attain a significant enrichment/depletion of SNP-heritability after correcting for multiple testing.

Functional category	% h_g^2	% h_g^2 standard error	% SNP	Enrichment	Enrichment standard error	p-value
Coding	3.0%	1.3%	1.8%	1.67	0.75	3.8E-01
UTR	1.6%	1.4%	1.9%	0.84	0.72	8.3E-01
Promoter	7.8%	1.8%	3.4%	2.32	0.54	1.5E-02
**LNCaP: H3k27ac**	**22.3%**	**2.1%**	**3.2%**	**6.90**	**0.64**	**2.5E-20**
ARBS	3.3%	1.1%	1.0%	3.21	1.10	4.5E-02
LNCaP: FOXA1	1.5%	1.3%	1.5%	1.05	0.88	9.5E-01
LNCaP: H3k4me1	1.3%	1.4%	2.0%	0.65	0.71	6.2E-01
LNCaP: DHS	5.4%	1.6%	2.9%	1.86	0.56	1.3E-01
DHS prostate	2.6%	1.4%	1.8%	1.49	0.81	5.4E-01
**DHS cancer**	**14.1%**	**2.3%**	**4.7%**	**2.97**	**0.48**	**3.9E-05**
H3k4me1 (other)	19.6%	3.5%	16.3%	1.20	0.21	3.5E-01
H3k27ac (other)	4.1%	2.4%	7.3%	0.57	0.33	1.9E-01
DHS (other)	0.2%	1.3%	1.8%	0.09	0.76	2.3E-01
**repressed**	**11.0%**	**4.1%**	**48.7%**	**0.23**	**0.08**	**2.1E-20**
all other	0.7%	1.2%	1.7%	0.38	0.69	3.7E-01

Supplementary Table 7. SNP-heritability estimates using variance components in the selected model using iCOGS typed data (no imputation) over 42,613 individuals. This selected model localized 51.0% of the SNP-heritability (h_g^2) within 12.1% of SNPs (LNCaP:H3K27ac + ARBS + DHS cancer).

**Functional category**	**% h_g^2**	**% h_g^2 standard error**	**% SNP**	**Enrichment**	**Enrichment standard error**	**p-value**
Coding	3.3%	1.4%	1.8%	1.88	0.77	2.6E-01
UTR	2.2%	1.4%	1.9%	1.15	0.73	8.3E-01
Promoter	8.5%	1.8%	3.4%	2.51	0.55	5.7E-03
LNCaP: H3K27ac	23.2%	2.1%	3.2%	7.19	0.65	*1.5E-21
ARBS	4.1%	1.2%	1.0%	3.98	1.14	9.0E-03
DHS cancer	23.7%	2.8%	7.9%	2.99	0.36	*2.1E-08
repressed	33.5%	4.1%	77.0%	0.43	0.05	*2.2E-26
all other	0.9%	1.6%	3.7%	0.25	0.42	7.2E-02

Supplementary Table 8. SNP-heritability estimates using variance components in the selected model using iCOGS imputed data over 42,613 individuals. This selected model localized 86% of the h_g^2 within 8.6% of SNPs (LNCaP:H3K27ac + ARBS + DHS cancer).

Functional category	% h_g^2	% h_g^2 standard error	% SNP	Enrichment	Enrichment standard error	p-value
Coding	0.9%	2.9%	0.7%	1.31	4.42	9.4E-01
UTR	3.0%	3.1%	1.0%	3.04	3.13	5.1E-01
Promoter	8.9%	4.1%	2.6%	3.41	1.58	1.3E-01
LNCaP: H3K27ac	27.0%	3.8%	2.8%	9.71	1.36	*1.5E-10
ARBS	9.1%	3.3%	0.6%	14.67	5.26	9.4E-03
DHS cancer	49.6%	6.3%	5.2%	9.56	1.22	*1.8E-12
repressed	0.3%	7.0%	82.8%	0.00	0.08	*1.0E-31
all other	0.2%	2.7%	4.4%	0.06	0.62	1.3E-01

Supplementary Table 9. SNP-heritability estimates using variance components in the selected model using iCOGS imputed data over 42,613 individuals. We further assessed the ARBS and LNCaP: H3K27ac functional categories in a hierarchical model in which imputed SNPs were first assigned to ARBS class (over LNCaP: H3K27ac). ARBS attained a highly significant 31-fold enrichment (as opposed to 15-fold in the model in which SNPs are first assigned to LNCaP: H3K27ac, see Supplementary Table 7). Consistent with Supplementary Table 7, this selected model localized 86% of the h_g^2 within 8.6% of SNPs (ARBS + LNCaP:H3K27ac + DHS cancer).

Functional category	% h_g^2	% h_g^2 standard error	% SNP	Enrichment	Enrichment standard error	p-value
Coding	0.6%	2.9%	0.7%	0.95	4.36	9.9E-01
UTR	3.0%	3.0%	1.0%	3.03	3.07	5.1E-01
Promoter	8.7%	4.0%	2.6%	3.34	1.55	1.3E-01
ARBS	26.5%	4.1%	0.9%	30.51	4.71	*3.7E-10
LNCaP: H3K27ac	14.9%	3.4%	2.5%	5.90	1.34	2.6E-04
DHS cancer	44.8%	6.1%	5.2%	8.62	1.18	*1.1E-10
repressed	0.2%	6.9%	82.8%	0.00	0.08	*6.6E-33
all other	0.4%	2.6%	4.4%	0.09	0.61	1.3E-01

Supplementary Table 10. SNP-heritability estimates using variance components in sub-categories of the selected model using iCOGS typed data (no imputation) over 42,613 individuals. To quantify the enrichment of SNP-heritability (h_g^2) in regions marked by both LNCaP: H3K27ac and ARBS, we partitioned the SNPs in LNCaP: H3K27ac and ARBS according to whether they are present in both categories or are exclusive to each. We observe that SNPs present in both LNCaP: H3K27ac and ARBS attain significantly higher enrichment (15.31-fold) than SNPs exclusive to each category (5.62-fold for LNCaP: H3K27ac and 4.05-fold for the ARBS specific categories).

Functional category	% h_g^2	% h_g^2 standard error	% SNP	Enrichment	Enrichment standard error	p-value
Coding	3.4%	1.4%	1.8%	1.91	0.80	2.5E-01
UTR	2.2%	1.4%	1.9%	1.14	0.75	8.5E-01
Promoter	8.6%	1.9%	3.4%	2.55	0.56	5.4E-03
LNCaP: H3K27ac (exclusively)	15.5%	1.9%	2.8%	5.62	0.68	1.0E-11
LNCaP: H3K27ac & ARBS (intersection)	6.8%	1.0%	0.4%	15.31	2.26	2.3E-10
ARBS (exclusively)	4.1%	1.2%	1.0%	4.05	1.16	8.5E-03
DHS cancer	24.7%	2.9%	7.9%	3.13	0.37	7.1E-09
repressed	32.4%	4.2%	76.7%	0.42	0.05	5.7E-26
all other	1.1%	1.6%	3.7%	0.30	0.43	1.0E-01

Supplementary Table 11. SNP-heritability estimates using variance components in the selected model using BPC3 European imputed data. We observe a high degree of correlation in the SNP-heritability (h_g^2) attributable to each functional category between the iCOGS (see Supplementary Table 7, main text) and BPC3 data sets. Analytical standard error on the estimate is higher in the BPC3 than iCOGS due to lower sample size. Annotations replicating significantly at P<0.05 are marked with '*'.

Functional category	% h_g^2	% h_g^2 standard error	% SNP	Enrichment	Enrichment standard error	p-value
Coding	0.2%	10.1%	0.6%	0.31	16.10	9.7E-01
UTR	21.0%	11.3%	0.8%	25.40	13.65	7.4E-02
Promoter	0.0%	12.7%	2.2%	0.00	5.71	8.6E-01
LNCaP: H3K27ac	30.3%	12.1%	2.6%	11.50	4.60	*2.3E-02
ARBS	1.1%	12.1%	0.6%	1.74	18.78	9.7E-01
DHS cancer	47.4%	21.4%	5.1%	9.27	4.19	*4.8E-02
repressed	0.0%	23.8%	84.3%	0.00	0.28	*4.0E-04
all other	0.0%	9.2%	3.6%	0.00	2.54	6.9E-01

Supplementary Table 12. SNP-heritability estimates using variance components in the selected model using AAPC African American imputed data. We observe a high degree of correlation in the SNP-heritability (h_g^2) attributable to each functional category between the iCOGS European (see Supplementary Table 7, main text) and AAPC African American data sets. Analytical standard error on the estimate is higher in AAPC than iCOGS due to lower sample size. Annotations replicating significantly at P<0.05 are marked with '*'.

Functional category	% h_g^2	% h_g^2 standard error	% SNP	Enrichment	Enrichment standard error	p-value
Coding	3.3%	11.1%	0.7%	4.93	16.53	8.1E-01
UTR	5.9%	11.2%	1.0%	6.05	11.45	6.6E-01
Promoter	0.0%	14.7%	2.7%	0.00	5.53	8.6E-01
LNCaP: H3K27ac	28.9%	12.7%	2.9%	9.98	4.39	*4.1E-02
ARBS	15.2%	12.1%	0.6%	23.66	18.73	2.3E-01
DHS cancer	46.6%	22.4%	5.5%	8.49	4.08	6.6E-02
repressed	0.0%	24.5%	86.6%	0.00	0.28	*4.2E-04
all other	0.0%	7.6%	4.4%	0.00	1.73	5.6E-01

Supplementary Table 13. SNP-heritability estimates using variance components in the selected model using BPC3 European imputed data at two levels of imputation accuracy. Filtering SNPs based on stringent imputation quality scores does not significantly change the SNP-heritability estimates for each functional category.

	**All markers**				**INFO > 0.9 markers**			
	**% h_g^2**	**% h_g^2 standard error**	**% SNP**	**p-value**	**% h_g^2**	**% h_g^2 standard error**	**% SNP**	**p-value**
Coding	0%	10%	1%	1E+00	1%	10%	1%	1E+00
UTR	21%	11%	1%	7E-02	18%	11%	1%	1E-01
Promoter	0%	13%	2%	9E-01	0%	12%	2%	9E-01
LNCaP: H3K27ac	30%	12%	3%	2E-02	30%	12%	3%	2E-02
ARBS	1%	12%	1%	1E+00	1%	12%	1%	1E+00
DHS cancer	47%	21%	5%	5E-02	50%	21%	5%	4E-02
repressed	0%	24%	84%	4E-04	0%	24%	87%	2E-04
all other	0%	9%	4%	7E-01	0%	9%	4%	7E-01

Supplementary Table 14. SNP-heritability estimates using variance components in the selected model using WTCCC European imputed data. We present results meta-analyzed across all 11 phenotypes: Ankylosing Spondylitis (AS); Bipolar Disorder (BD); Coronary Artery Disease (CAD); Crohn's Disease (CD); Hypertension (HT); Multiple Sclerosis (MS); Rheumatoid Arthritis (RA); Schizophrenia (SP); Type 1 Diabetes (T1D); Type 2 Diabetes (T2D); Ulcerative Colitis (UC). After quality control, a total of 47,053 samples and 4-5 million genotyped and imputed SNPs remained. Reported h_g^2 values were estimated for each phenotype separately and meta-analyzed using inverse-variance weighting.

Functional category	% h_g^2	% h_g^2 standard error	% SNP	Enrichment	Enrichment standard error	p-value
Coding	2.2%	1.6%	0.5%	4.11	2.86	2.8E-01
UTR	3.3%	1.6%	0.8%	4.21	2.12	1.3E-01
Promoter	2.3%	2.1%	2.2%	1.05	0.97	9.6E-01
LNCaP: H3K27ac	1.1%	1.6%	2.6%	0.43	0.59	3.4E-01
ARBS	11.6%	1.9%	0.6%	19.17	3.20	*1.4E-08
DHS cancer	47.5%	3.6%	4.8%	9.82	0.74	*4.0E-33
repressed	28.1%	3.6%	87.8%	0.32	0.04	*4.4E-63
all other	2.8%	1.5%	3.7%	0.75	0.39	5.2E-01

Supplementary Table 15. Risk prediction accuracy in cross-validations from iCOGS data. All predictions were carried out by cross-validation in the iCOGS data, removing 1,000 individuals in each fold. Prediction R^2 was then computed from a regression of phenotype on the predictor score with 10 PCs included as covariates to account for ancestry, subsequently subtracting the R^2=0.021 from a model with PCs only. To ensure that prediction across data sets was independent, we carefully removed all iCOGS individuals with a GRM value of >0.05 to any individual in the BPC3 when computing BLUP coefficients. We separately analyzed the predictor in 26,000 iCOGS samples that had age at diagnosis, but did not observe significant differences before/after including age as a covariate. We report coefficient-specific P-values from a multiple regression of phenotype ~ top SNPs score + single-BLUP score + multi-BLUP score + PCs.

**Predictor**	**Prediction R^2**	**p-value in joint model**
top SNPs	0.029	*NA*
single BLUP	0.065	*NA*
multi BLUP	0.071	*NA*
top SNPs + single BLUP	0.069	2.3E-06
top SNPs + multi BLUP	0.074	5.3E-31
known SNPs [*Al Aloma et al. Nat. Gen. 2014*]	0.084	*NA*
known SNPs + single BLUP	0.096	6.7E-04
known SNPs + multi BLUP	0.098	1.3E-23

Supplementary Table 16. SNP-heritability estimates using variance components partitioned by minor allele frequency using iCOGS imputed data over 42,613 individuals. We observe that most of the SNP-heritability in the iCOGS data comes from common variation. P-value reported for difference between % h_g^2 and % SNP.

Frequency Bin	% h_g^2	% h_g^2 standard error	% SNP	p-value
1-5%	6%	2%	7%	7E-01
5-12%	17%	3%	19%	6E-01
12-20%	13%	3%	19%	4E-02
20-29%	19%	3%	19%	8E-01
29-39%	18%	3%	19%	8E-01
39-50%	26%	3%	19%	7E-03

Supplementary Note 1: Additional acknowledgements

We would like to acknowledge the contribution of the Australian Prostate Cancer Research Centre-Qld, Institute of Health and Biomedical Innovation and School of Biomedical Science, Translational Research Institute, Queensland University of Technology, Brisbane, Australia. The Australian Prostate Cancer BioResource is contributed by following author other than those named in the author list

Prof Gail Risbridger[1,2], Dr Renea Taylor[1,2], Prof Wayne Tilley[3,4], A/Prof Lisa Butler[3,4], A/Prof Lisa Horvath[5,6,7], Dr Trina Yeadon[8,9,10], Ms Allison Eckert[8,9,10], Dr Glen Wood[11], Dr Peter Heathcote[11], Dr Greg Malone[11], Dr Kris Kerr[12], Dr Megan Turner[12], Dr Angus Collins[12]

1. Prostate Cancer Research Group, Monash University, Clayton, VIC, 2. Australian Prostate Cancer BioResource, Melbourne, VIC, 3. Adelaide Prostate Cancer Research Centre, Adelaide, SA, 4. Australian Prostate Cancer BioResource, Adelaide, SA, 5. Garvan Institute of Medical Research/The Kinghorn Cancer Centre, Darlinghurst, NSW, 6. Chris O'Brien Lifehouse, Camperdown, NSW, 7. Australian Prostate Cancer BioResource, Sydney, NSW, 8. Cancer Program, Institute of Health and Biomedical Innovation, Queensland University of Technology, Brisbane, QLD, 9. Australian Prostate Cancer Research Centre-Queensland at the Princess Alexandra Hospital, Queensland University of Technology, Brisbane, QLD, 10. Australian Prostate Cancer BioResource, Brisbane, QLD, 11. The Brisbane Urology Clinic Central Queensland Urology Clinic, QLD, 12. Sullivan and Nicolaides Pathology, Brisbane, QLD.

This work was supported by the Canadian Institutes of Health Research, European Commission's Seventh Framework Programme grant agreement n° 223175 (HEALTH-F2-2009-223175), Cancer Research UK Grants C5047/A7357, C1287/A10118, C5047/A3354, C5047/A10692, C16913/A6135, and The National Institute of Health (NIH) Cancer Post-Cancer GWAS initiative grant: No. 1 U19 CA 148537-01 (the GAME-ON initiative). This study would not have been possible without the contributions of the following: Per Hall (COGS); Douglas F. Easton, Paul Pharoah, Kyriaki Michailidou, Manjeet K. Bolla, Qin Wang (BCAC), Andrew Berchuck (OCAC), Rosalind A. Eeles, Douglas F. Easton, Ali Amin Al Olama, Zsofia Kote-Jarai, Sara Benlloch (PRACTICAL), Georgia Chenevix-Trench, Antonis Antoniou, Lesley McGuffog, Fergus Couch and Ken Offit (CIMBA), Joe Dennis, Alison M. Dunning, Andrew Lee, and Ed Dicks, Craig Luccarini and the staff of the Centre for Genetic Epidemiology Laboratory, Javier Benitez, Anna Gonzalez-Neira and the staff of the CNIO genotyping unit, Jacques Simard and Daniel C. Tessier, Francois Bacot, Daniel Vincent, Sylvie LaBoissière and Frederic Robidoux and the staff of the McGill University and Génome Québec Innovation Centre, Stig E. Bojesen, Sune F. Nielsen, Borge G. Nordestgaard, and the staff of the Copenhagen DNA laboratory, and Julie M. Cunningham, Sharon A. Windebank, Christopher A. Hilker, Jeffrey Meyer and the staff of Mayo Clinic Genotyping Core Facility. Funding for the iCOGS infrastructure came from: the European Community's Seventh Framework Programme under grant agreement n° 223175 (HEALTH-F2-2009-223175) (COGS), Cancer Research UK (C1287/A10118, C1287/A 10710, C12292/A11174, C1281/A12014, C5047/A8384, C5047/A15007, C5047/A10692), the National Institutes of Health (CA128978) and Post-Cancer GWAS initiative (1U19 CA148537, 1U19 CA148065 and 1U19 CA148112 - the GAME-ON initiative), the Department of Defence (W81XWH-10-1-0341), the Canadian Institutes of Health Research (CIHR) for the CIHR Team in Familial Risks of Breast Cancer, Komen Foundation for the Cure, the Breast Cancer Research Foundation, and the Ovarian Cancer Research Fund. The Department of Medical Epidemiology and Biostatistics, Karolinska Institute, Stockholm, Sweden was supported by the Cancer Risk Prediction Center (CRisP; www.crispcenter.org), a Linneus Centre (Contract ID 70867902) financed by the Swedish Research Council, Swedish Research Council (grant no K2010-70X-20430-04-3), the Swedish Cancer Foundation (grant no 09-0677), the Hedlund Foundation, the Soederberg Foundation, the Enqvist Foundation, ALF funds from the Stockholm County Council. Stiftelsen Johanna Hagstrand och Sigfrid Linner's Minne, Karlsson's Fund for urological and surgical research. We thank and acknowledge all of the participants in the Stockholm-1 study. We thank Carin Cavalli-Bjoerkman and Ami Roennberg Karlsson for their dedicated work in the collection of data. Michael Broms is acknowledged for his skilful work with the databases. KI Biobank is acknowledged for

handling the samples and for DNA extraction. Hans Wallinder at Aleris Medilab and Sven Gustafsson at Karolinska University Laboratory are thanked for their good cooperation in providing historical laboratory results. The ESTHER study was supported by a grant from the Baden Württemberg Ministry of Science, Research and Arts. Additional cases were recruited in the context of the VERDI study, which was supported by a grant from the German Cancer Aid (Deutsche Krebshilfe). The ESTHER group would like to thank Hartwig Ziegler, Sonja Wolf, Volker Hermann, Katja Butterbach. The FHCRC studies were supported by grants RO1CA056678, RO1CA082664, and RO1CA092579 from the US National Cancer Institute, National Institutes of Health, with additional support from the Fred Hutchinson Cancer Research Center. The IPO-Porto study was in part funded by Liga Portuguesa Contra o Cancro. The Mayo group was supported by the US National Cancer Institute (R01CA72818) The Prostate Cancer Program of Cancer Council Victoria also acknowledge grant support from The National Health and Medical Research Council, Australia (126402, 209057, 251533, , 396414, 450104, 504700, 504702, 504715, 623204, 940394, 614296,), VicHealth, Cancer Council Victoria, The Prostate Cancer Foundation of Australia, The Whitten Foundation, PricewaterhouseCoopers, and Tattersallï¾'s. EAO, DMK, and EMK acknowledge the Intramural Program of the National Human Genome Research Institute for their support. The MEC was support by NIH grants CA63464, CA54281 and CA098758. The Moffitt group was supported by the US National Cancer Institute (R01CA128813, PI: J.Y. Park). The PCMUS study was supported by the Bulgarian National Science Fund, Ministry of Education and Science (contract DOO-119/2009; DUNK01/2-2009; DFNI-B01/28/2012) with additional support from the Science Fund of Medical University - Sofia (contract 51/2009; 8I/2009; 28/2010). ProtecT would like to acknowledge the support of The University of Cambridge, Cancer Research UK. Cancer Research UK grants [C8197/A10123] and [C8197/A10865] supported the genotyping team. We would also like to acknowledge the support of the National Institute for Health Research which funds the Cambridge Bio-medical Research Centre, Cambridge, UK. We would also like to acknowledge the support of the National Cancer Research Prostate Cancer: Mechanisms of Progression and Treatment (PROMPT) collaborative (grant code G0500966/75466) which has funded tissue and urine collections in Cambridge. We are grateful to staff at the Welcome Trust Clinical Research Facility, Addenbrookeï¾'s Clinical Research Centre, Cambridge, UK for their help in conducting the ProtecT study. We also acknowledge the support of the NIHR Cambridge Biomedical Research Centre, the DOH HTA (ProtecT grant) and the NCRI / MRC (ProMPT grant) for help with the bio-repository. The UK Department of Health funded the ProtecT study through the NIHR Health Technology Assessment Programme (projects 96/20/06, 96/20/99). The ProtecT trial and its linked ProMPT and CAP (Comparison Arm for ProtecT) studies are supported by Department of Health, England; Cancer Research UK grant number C522/A8649, Medical Research Council of England grant number G0500966, ID 75466 and The NCRI, UK. The epidemiological data for ProtecT were generated though funding from the Southwest National Health Service Research and Development. DNA extraction in ProtecT was supported by USA Dept of Defense award W81XWH-04-1-0280, Yorkshire Cancer Research and Cancer Research UK. The authors would like to acknowledge the contribution of all members of the ProtecT study research group. The views and opinions expressed therein are those of the authors and do not necessarily reflect those of the Department of Health of England. The bio-repository from ProtecT is supported by the NCRI (ProMPT) Prostate Cancer Collaborative and the Cambridge BMRC grant from NIHR. The QLD research is supported by The National Health and Medical Research Council, Australia Project Grant [390130, 1009458] and Enabling Grant [614296 to APCB]; the Prostate Cancer Foundation of Australia (Project Grant [PG7] and Research infrastructure grant [to APCB]). SCCS is funded by NIH grant R01 CA092447, and SCCS sample preparation was conducted at the Epidemiology Biospecimen Core Lab that is supported in part by the Vanderbilt-Ingram Cancer Center (P30 CA68485). Data on SCCS cancer cases used in this publication were provided by the Alabama Statewide Cancer Registry; Kentucky Cancer Registry, Lexington, KY; Tennessee Department of Health, Office of Cancer Surveillance; Florida Cancer Data System; North Carolina Central Cancer Registry, North Carolina Division of Public Health; Georgia Comprehensive Cancer Registry; Louisiana Tumor Registry; Mississippi Cancer Registry; South Carolina Central Cancer Registry; Virginia Department of Health, Virginia Cancer Registry; Arkansas Department of Health, Cancer Registry, 4815 W. Markham, Little Rock, AR 72205. The Arkansas Central Cancer Registry is fully funded by a grant from National Program of Cancer Registries, Centers for Disease Control and Prevention (CDC). Data on SCCS cancer cases from Mississippi were collected by the Mississippi Cancer Registry which participates in the National Program of Cancer Registries (NPCR) of

the Centers for Disease Control and Prevention (CDC). The contents of this publication are solely the responsibility of the authors and do not necessarily represent the official views of the CDC or the Mississippi Cancer Registry. SEARCH is funded by a programme grant from Cancer Research UK [C490/A10124] and supported by the UK National Institute for Health Research Biomedical Research Centre at the University of Cambridge. The Tampere (Finland) study was supported by the Academy of Finland (251074), The Finnish Cancer Organisations, Sigrid Juselius Foundation, and the Competitive Research Funding of the Tampere University Hospital (9N069 and X51003). The PSA screening samples were collected by the Finnish part of ERSPC (European Study of Screening for Prostate Cancer). Riina Liikanen is thanked for technical assistance. Riitta Vaalavuo and Liisa Maeaettaenen are thanked for their work with databases. UKGPCS would also like to thank the following for funding support: The Institute of Cancer Research and The Everyman Campaign, The Prostate Cancer Research Foundation, Prostate Research Campaign UK (now Prostate Action), The Orchid Cancer Appeal, The National Cancer Research Network UK, The National Cancer Research Institute (NCRI) UK. We are grateful for support of NIHR funding to the NIHR Biomedical Research Centre at The Institute of Cancer Research and The Royal Marsden NHS Foundation Trust. UKGPCS should also like to acknowledge the NCRN nurses, data managers and Consultants for their work in the UKGPCS study. UKGPCS would like to thank all urologists and other persons involved in the planning, coordination, and data collection of the CAPS study. The Ulm group received funds from the German Cancer Aid (Deutsche Krebshilfe). The Keith and Susan Warshaw Fund, C. S. Watkins Urologic Cancer Fund and The Tennity Family Fund supported the Utah study. The project was supported by Award Number P30CA042014 from the National Cancer Institute

The coordination of EPIC is financially supported by the European Commission (DG-SANCO) and the International Agency for
Research on Cancer. The national cohorts (that recruited male participants) are supported by Danish Cancer Society (Denmark); German Cancer Aid, German Cancer Research Center (DKFZ), Federal Ministry of Education and Research (BMBF), Deutsche Krebshilfe, Deutsches Krebsforschungszentrum and Federal Ministry of Education and Research (Germany); the Hellenic Health Foundation (Greece); Associazione Italiana per la Ricerca sul Cancro-AIRC-Italy and National Research Council (Italy); Dutch Ministry of Public Health, Welfare and Sports (VWS), Netherlands Cancer Registry (NKR), LK Research Funds, Dutch Prevention Funds, Dutch ZON (Zorg Onderzoek Nederland), World Cancer Research Fund (WCRF), Statistics Netherlands (The Netherlands); Health Research Fund (FIS), PI13/00061 to Granada; , PI13/01162 to EPIC-Murcia), Regional Governments of Andalucía, Asturias, Basque Country, Murcia and Navarra, ISCIII RETIC (RD06/0020) (Spain); Swedish Cancer Society, Swedish Research Council and County Councils of Skåne and Västerbotten (Sweden); Cancer Research UK (14136 to EPIC-Norfolk; C570/A16491 and C8221/A19170 to EPIC-Oxford), Medical Research Council (1000143 to EPIC-Norfolk, MR/M012190/1 to EPIC-Oxford) (United Kingdom).

Additional information for the PRACTICAL consortium can be found at http://practical.ccge.medschl.cam.ac.uk/
